# Identification of methylation changes associated with positive and negative growth deviance in Gambian infants using a targeted methyl sequencing approach of genomic DNA

**DOI:** 10.1096/fba.2020-00101

**Published:** 2021-02-05

**Authors:** Claire R. Quilter, Kerry M. Harvey, Julien Bauer, Benjamin M. Skinner, Maria Gomez, Manu Shrivastava, Andrew M. Doel, Saikou Drammeh, David B. Dunger, Sophie E. Moore, Ken K. Ong, Andrew M. Prentice, Robin M. Bernstein, Carole A. Sargent, Nabeel A. Affara

**Affiliations:** ^1^ Department of Pathology University of Cambridge Cambridge UK; ^2^ School of Life Sciences University of Essex Colchester UK; ^3^ Department of Women and Children's Health King's College London London UK; ^4^ MRC Unit The Gambia at London School of Hygiene and Tropical Medicine Banjul The Gambia; ^5^ Growth and Development Lab Department of Anthropology University of Colorado Boulder CO USA; ^6^ MRC Epidemiology Unit University of Cambridge School of Clinical Medicine Cambridge UK; ^7^ Department of Paediatrics University of Cambridge School of Clinical Medicine Cambridge UK; ^8^ Institute of Metabolic Science Cambridge Biomedical Campus Cambridge Cambridge UK; ^9^ Institute of Behavioural Science University of Colorado Boulder CO USA; ^10^ Present address: East Midlands & East of England NHS Genomic Laboratory Hub, Genomics Laboratories Cambridge University Hospitals NHS Foundation Trust Cambridge UK; ^11^ Present address: Kennedy Institute of Rheumatology University of Oxford Oxford UK; ^12^ Present address: Oxford University Hospitals Oxford UK

**Keywords:** birthweight, DNA methylation, environmental exposures, stunting

## Abstract

Low birthweight and reduced height gain during infancy (stunting) may arise at least in part from adverse early life environments that trigger epigenetic reprogramming that may favor survival. We examined differential DNA methylation patterns using targeted methyl sequencing of regions regulating gene activity in groups of rural Gambian infants: (a) low and high birthweight (DNA from cord blood (*n* = 16 and *n* = 20, respectively), from placental trophoblast tissue (*n* = 21 and *n* = 20, respectively), and DNA from peripheral blood collected from infants at 12 months of age (*n* = 23 and *n* = 17, respectively)), and, (b) the top 10% showing rapid postnatal length gain (high, *n* = 20) and the bottom 10% showing slow postnatal length gain (low, *n* = 20) based on z score change between birth and 12 months of age (LAZ) (DNA from peripheral blood collected from infants at 12 months of age). Using BiSeq analysis to identify significant methylation marks, for birthweight, four differentially methylated regions (DMRs) were identified in trophoblast DNA, compared to 68 DMRs in cord blood DNA, and 54 DMRs in 12‐month peripheral blood DNA. Twenty‐five DMRs were observed to be associated with high and low length for age (LAZ) at 12 months. With the exception of five loci (associated with two different genes), there was no overlap between these groups of methylation marks. Of the 194 CpG methylation marks contained within DMRs, 106 were located to defined gene regulatory elements (promoters, CTCF‐binding sites, transcription factor‐binding sites, and enhancers), 58 to gene bodies (introns or exons), and 30 to intergenic DNA. Distinct methylation patterns associated with birthweight between comparison groups were observed in DNA collected at birth (at the end of intrauterine growth window) compared to those established by 12 months (near the infancy/childhood growth transition). The longitudinal differences in methylation patterns may arise from methylation adjustments, changes in cellular composition of blood or both that continue during the critical postnatal growth period, and in response to early nutritional and infectious environmental exposures with impacts on growth and longer‐term health outcomes.

**Abbreviations**
Cis‐eQTM: Cis‐acting quantitative trait methylation
Cis‐meQTL: Cis‐acting methylation quantitative trait locus
CTCF: CCCTC‐binding factor
DAVID: Database for Annotation, Visualization, and Integrated Discovery
DMR: Differentially Methylated Region
EBI: European Bioinformatics Institute
EWAS: Epigenome‐Wide Association Study
FDR: False Discovery Rate
GAD: Genetic Association Database
GWAS: Genome‐Wide Association Study
LAZ: Length for Age Z score
MAF: Minor Allele Frequency
MRC: Medical Research Council
NCBI: National Center for Bioinformatics Technology
OMIM: Online Mendelian Inheritance in Man
PCA: Principal Components Analysis
SGA: Small for Gestation Age
SNP: Single Nucleotide Polymorphism
Trans‐meQTL: Trans‐acting methylation quantitative trait locus
TSS: Transcription Start Site
UGR: Uterine Growth Restriction

## 1 INTRODUCTION

About 45% of global deaths in children under 5 years of age are thought to be related to undernutrition.^1^ Children who survive early periods of undernutrition may suffer longer‐term consequences, including stunting and other developmental deficits,^2^ which are major contributors to long‐term morbidity and mortality.^3^, ^4^ Although the prevalence of stunting declined in sub‐Saharan Africa from 42% in 1990 to 32% in 2015, the numbers of affected individuals increased from 47 million to 58 million.^5^ Studies estimate that 20% of growth retardation starts *in utero* where under‐nutrition in pregnancy increases the risks of intrauterine growth retardation (IUGR) and small for gestation age (SGA) infants, preterm delivery^6^ and long‐term impaired immunity. It is hypothesized that an adverse early life environment and nutrition induce phenotypic adaptations through developmental plasticity^7^ to favor survival in the short term, but at the expense of lifelong effects on health.^8^, ^9^

Nutritional interventions to improve child growth and adult health^10^, ^11^ have had limited success, primarily for the lack of a clear understanding of optimal timing, target groups, and the composition of supplements. The period of growth and development from conception to a child's second birthday (coined the first 1000 days) is one of the most critical windows of opportunity for interventions.^12^ There is a complex interplay between an individual's genetic constitution and the environment. Responses to extrinsic factors via modifications to the epigenome (which may include to both chromatin‐associated proteins and DNA bases) in the first 1000 days are believed to be important in establishing protective adaptations against the impact of under‐nutrition and an adverse environment (thrifty phenotype).^13^, ^14^ DNA methylation at CpG couplets is one of the most actively studied modifications to the epigenome.

A large meta‐analysis of multiple epigenome‐wide association studies (EWAS) by the Childhood Epigenetics Consortium found methylation at 914 CpG sites associated with birthweight in whole blood DNA from healthy neonates, but <1.3% persisted in children (2–13 years), <0.1% in adolescents (16–18 years), and none in adults (30–45 years).^15^ The current study uses samples and data from a cohort of Gambian mother–infant pairs exhibiting high rates of maternal and child under‐nutrition. Rural Gambian infants are small at birth relative to international standards, show positive growth patterns during the first few months of life and then, enter a period of reduced growth marked by profound faltering until at least 24 months of age.^16^, ^17^ Schoenbuchner et al.^16^ have suggested that stunting is an extreme adaptation to profound faltering episodes potentially arising from a complex interaction of malnutrition, infection, and disease. Despite four decades of nutrition‐sensitive and nutrition‐specific interventions halving under‐nutrition for young children from rural Gambia, substantial (30%) growth faltering remains,^17^ indicating a gap in our understanding of its complex etiology.

Epigenetic studies carried out on Gambian populations have highlighted the importance of maternal nutrition and exposures and the effects of maternal nutritional supplementation in this highly seasonal environment. Many aspects of health and behavior in rural Gambia are influenced by the annual seasonality with a single rainy “hungry” season (late June–October) followed by a dry “harvest” season (November–May/June).^2^, ^18^ Specifically, there is evidence that seasonal variation in nutrition during the periconceptional period influences methylation status in postnatal infants at a number of loci,^19^ is related to methyl‐donor nutrient content of the mother's diet ^20^, ^21^, ^22^, ^23^ and may be associated with an increase in both preterm and SGA infants.^18^ Periconceptional nutrition supplementation influences methylation changes in cord and postnatal infant blood DNA at CpG loci linked to genes associated with infection and immunity^24^ and alters the methylation at imprinted loci.^25^ Maternal exposure to aflatoxin B1 is also associated with DNA methylation changes at specific loci in Gambian infants.^26^

The aim of the present study was to identify epigenetic marks that are established during the critical first 1000 days in a cohort of rural Gambian infants and explore how these may be associated with normal versus stunted growth outcomes in order to determine whether any targets for intervention are associated with prenatal and/or postnatal periods of epigenetic modification. We used a targeted methyl sequencing approach of genomic DNA from placental trophoblast tissue, cord, and infant (12 months of age) blood to identify the methylation changes. These changes may be useful as biomarkers, highlighting genes influenced by exposures during embryonic and fetal development and early infancy, and identifying potential pathways through which these may influence the growth outcomes at birth and in the first year of life.

## 2 MATERIALS AND METHODS

### 2.1 Samples

The study was conducted among pregnant women and their infants living in the rural West Kiang region of The Gambia. Participants were recruited as part of the HERO‐G (Hormonal Regulators of Growth) study. The study cohort was 238 newborns whose growth had been assessed longitudinally to 24 months of age. Table 1 summarizes data associated with the samples from individuals used in this study. The full HERO‐G protocol is described elsewhere.^27^ Placentas from women who delivered at home were collected by trained field workers and immediately transported on ice to the nearby Medical Research Council (MRC) Unit The Gambia Keneba laboratory (within 20–30 minutes) and carefully processed to obtain trophoblast material following a standard protocol (see placenta sample collection protocol in Data S1). Placental samples each of 400 mg were taken at four different evenly spaced locations, at least 2 cm from the edge, and at consistent relative positions in each placenta to mitigate placental tissue heterogeneity. Samples were cut into four pieces, placed in RNAlater at a volume of 5 x tissue weight (Cat No 76106, Qiagen), and transported frozen on dry‐ice to the United Kingdom for DNA extraction. After extraction samples from each of the four placental regions were pooled equimolarly. Cord blood and infant blood samples were collected into EDTA‐lined tubes (BD Vacutainer, pink top) for DNA extraction in the United Kingdom. Ethical approval for the study was given by the joint Gambia Government/Medical Research Council (MRC) Unit The Gambia Ethics Committee (SCC 1313v3), with additional approval from the University of Colorado Institutional Research Board (protocol number 13–0441). Community approval was obtained from each participating village, and written, informed consent was obtained from each participating family. Samples for analysis were selected retrospectively from the study cohort representing (a) the highest 20% and lowest 20% birthweights and (b) according to the top and bottom 10% change in length‐for‐age (LAZ) from birth to 12 months. For the 12‐month samples the male average age = 376.4 days, SD 9 days (366–409 d) and females average age = 378.8 days, SD 10 days (367–413 d). Table 2 summarizes the number of samples analyzed after quality testing for each tissue and test group and those that are common between groups.

**TABLE 1:** Summary of Individual‐Specific Data of Those Included in the Study

Mat ID	Mat Age	GA (wks)	Parity (Cat)	S_MOC	S_MOB	BW (kg)	BW Cat	BL (cm)	LAZ Change V1–12 m	LAZ Cat	Tissue
MALES											
	20.27	36	Primiparous	W	W	1.7	low	45.00	−0.45	low	Pl, CB, 12mB, 12mH
	35.19	38.1	Multiparous	D	D	2.14	low	44.43	1.55	high	Pl, 12mB, 12mH,
	33.32	38.1	Multiparous	D	D	2.32	low	47.00	0.53	mid	Pl, CB, 12mB
	38.61	38.6	Multiparous	W	D	2.38	low	45.50	−0.39	low	Pl, CB, 12mB, 12mH
	23.6	37	Primiparous	W	D	2.44	low	47.80	0.42	mid	Pl, CB, 12mB
	23.32	37.8	Multiparous	W	D	2.48	low	45.90	0	mid	Pl
	26.33	40.7	Multiparous	D	D	2.51	low	49.00	2.14	high	CB,12mB, 12mH
	27	39.9	Multiparous	W	W	2.53	low	49.00	1.7	high	Pl, CB, 12mB, 12mH
	24.39	38.9	Multiparous	D	D	2.56	low	47.40	0.99	high	Pl, CB, 12mB
	18.65	39.4	Primiparous	D	W	2.59	low	50.40	0.5	mid	Pl, CB, 12mB
	31.49	41.2	Multiparous	W	D	2.59	low	42.30	−0.49	low	Pl, 12mB, 12mH
	20.67	38.9	Primiparous	D	D	2.67	low	46.70	1.8	high	12mB, 12mH
	31.16	40.7	Multiparous	D	D	2.7	low	48.40	1.21	high	12mB, 12mH
		45		W	D	2.91	mid		0.97	high	12mH
	38.5	41	Multiparous	D	W	2.92	mid	49.00	−0.91	low	12mH
	40.82	39.7	Multiparous	D	W	2.96	mid	50.10	1.96	high	12mH
	28.69	40.7	Multiparous	D	D	3.06	mid	49.33	−0.43	low	12mH
	41.27	40.7	Multiparous	D	D	3.07	mid	52.00	−0.75	low	12mH
	32.82	39.9	Multiparous	W	D	3.13	mid	53.00	−0.66	low	12mH
	27.11	41.2	Multiparous	D	D	3.26	high	51.50	−0.49	low	12mH
	23.04	39.4	Multiparous	W	D	3.26	high	51.23	−0.91	low	12mH
	29.15	38.6	Multiparous	D	D	3.26	high	48.00	1.52	high	12mB, 12mH
	37	38.1	Multiparous	D	D	3.27	high	48.10	1.37	high	12mB, 12mH
	37.53	38.1	Multiparous	W	D	3.28	high	49.30	1.08	high	Pl, CB, 12mB, 12mH
	25.64	40.2	Multiparous	D	W	3.34	high	51.00	0.95	mid	Pl, CB, 12mB
	34.46	40.4	Multiparous	W	W	3.34	high	48.30	0.19	mid	Pl, CB, 12mB
	20.29	40.2	Multiparous	W	W	3.36	high	53.47	−1.01	low	Pl, CB, 12mB, 12mH
	37.18	39.1	Multiparous	D	D	3.36	high	50.50	1.82	high	Pl, CB, 12mB, 12mH
	22.07	41	Multiparous	D	W	3.37	high	48.47	0	mid	Pl, CB
	28.91	40.2	Multiparous	D	D	3.39	high	50.50	0	mid	Pl, CB
	23.51	38.9	Multiparous	D	W	3.45	high	50.47	1.02	high	Pl
	31.48	40.7	Multiparous	D	D	3.5	high	50.00	0.08	mid	12mB
	37.96	41.2	Multiparous	D	D	3.52	high	50.40	1.7	high	12mB, 12mH
	40.36	39.1	Multiparous	W	D	3.55	high	49.50	0.6	mid	Pl
	41.88	41	Multiparous	D	W	3.59	high	51.00	−0.33	mid	Pl, CB, 12mB
	39.33	40.7	Multiparous	D	D	3.72	high	52.50	−0.93	low	Pl, CB
	31.19	41.8	Multiparous	D	D	3.79	high	50.77	−0.28	mid	Pl, CB, 12mB
	39.69	40.2	Multiparous	D	W	3.8	high	53.00	−1.35	low	Pl, CB, 12mB, 12mH
	39.65	41	Multiparous	D	W	3.9	high	50.00	0.82	mid	Pl, CB, 12mB
	36.61		Multiparous	D	W				1.31	high	12mH
FEMALES											
	18.72	39.4	Primiparous	D	W	2.42	low	46.00	1.12	high	Pl, 12mB, 12mH,
	19.04	39.9	Primiparous	W	W	2.46	low	46.00	0.34	mid	Pl, CB, 12mB
	29.89	39.1	Multiparous	D	D	2.48	low	50.00	0.54	mid	Pl, CB
	21.16	38.9	Multiparous	D	D	2.48	low	45.50	1.86	high	12mB, 12mH
	23.01	38.7	Multiparous	D	D	2.5	low	47.00	0.11	mid	12mB
	39.72	38.6	Multiparous	D	W	2.53	low	46.40	−0.28	mid	Pl, 12mB
	22.98	38.3	Multiparous	D	D	2.54	low	49.97	1.14	high	Pl, CB, 12mB, 12mH
	38.38	39.7	Multiparous	W	D	2.56	low	47.37	0.05	mid	Pl, CB, 12mB
	29.65	40.2	Multiparous	W	D	2.58	low	45.00	0	mid	Pl, CB
	26.92	38.1	Multiparous	W	D	2.61	low	46.50	0.65	mid	Pl, CB, 12mB
	41.69	38.1	Multiparous	D	D	2.63	low	48.50	0.9	mid	Pl, 12mB
	40.49	37	Multiparous	W	W	2.64	low	47.20	1.15	high	Pl, CB, 12mB, 12mH
	19.19	38.1	Primiparous	W	W	2.67	low	46.50	−4.44	low	Pl, CB, 12mB
	38.56	38.9	Multiparous	D	W	2.69	low	45.67	1.44	high	12mH
	32.99	40.7	Multiparous	D	D	2.73	low	46.27	−0.39	low	12mH
	23.95	39.9	Multiparous	W	W	2.96	mid	50.27	−0.47	low	12mH
	22.03	42	Multiparous	D	D	2.96	mid	49.40	1.67	high	12mH
	37.22	39.4	Multiparous	W	D	2.99	mid	49.50	−0.46	low	12mH
	26.08	38.3	Multiparous	D	D	3	mid	46.00	−0.46	low	12mH
	36.2	41.2	Multiparous	D	D	3.07	mid	50.07	−0.89	low	12mH
	38.07	40.4	Multiparous	D	D	3.21	mid	50.27	−0.76	low	12mH
	34.8	38.9	Multiparous	W	D	3.25	high	53.20	1.1	high	CB
	35.4	40.4	Multiparous	D	D	3.29	high	50.50	0.78	mid	CB
	36.78	40.2	Multiparous	D	W	3.31	high	49.00	0.07	mid	Pl
	32.43	39.7	Multiparous	W	D	3.33	high	51.00	0.81	mid	Pl, CB
	24.1	39.9	Multiparous	W	D	3.33	high	50.20	0	mid	CB
	34.3	39.4	Multiparous	W	D	3.33	high	50.00	−0.67	low	CB
	34.25	41	Multiparous	W	D	3.37	high	45.17	−1.39	low	Pl, CB, 12mB, 12mH
	39.54	40.2	Multiparous	W	D	3.42	high	49.80	−1.11	low	12mB, 12mH
	27.27	39.9	Multiparous	D	D	3.75	high	53.00	−0.17	mid	Pl, CB
	38.38	40.7	Multiparous	D	D	3.84	high	47.50	0.97	high	Pl, CB, 12mB
	27.12	41	Multiparous	D	D	3.97	high	52.00	−0.87	low	Pl, 12mB, 12mH
	27.07		Multiparous	D	W				1.1	high	12mH

Summary of individual‐specific data for subjects contributing to study. Key: Mat Age, Maternal age; GA, Gestational age; S_MOC, Season of month of conception; S_MOB, Season of month of birth; BW, Birthweight; LAZ, Length for age Z score change birth to 12 months; BL, Birth length; D, Dry season; W, Wet season; pl, Placenta; CB, Cord blood; 12 mB, 12‐month blood sample selected on birthweight; and 12 mH, 12‐month blood sample selected on LAZ score. The samples categorized as high or low for both birthweight and length for age were those used in the analysis. Table 2 shows the numbers and sex for each tissue.

**TABLE 2:** Summary of DNA Samples Analyzed in the Study

	DNA extraction		
	Low BW	High BW	Total
Placenta			
Male	10	14	24
Female	11	6	17
Both	21	20	**41**
Cord bloods			
Male	8	12	20
Female	8	8	16
Both	16	20	**36**

	DNA extraction					
	Low BW	High BW	Total	Low LAZ	High LAZ	Total
Infant blood (12 m)						
Male	12	13	25	11	13	24
Female	11	4	15	9	7	16
Both	23	17	**40**	20	20	**40**
Subjects in Common						
Placenta BW and cord blood BW	30					
Cord blood BW and Infant blood (12 m) BW	26					
Cord blood BW and Infant blood (12 m) LAZ	11					
Infant blood (12 m) BW and Infant blood (12 m) LAZ	22					

Numbers of DNA samples analyzed for each tissue according to sex and test group and the number of subjects in common between tissues and test groups. Key: BW, Birthweight; LAZ, Length for age Z score.

### 2.2 Nucleic acid extraction

DNA for DNA methylation studies was extracted from tissues using the Quick‐DNA Mini Prep Plus kit (Cat No. D4068, Zymo Research). DNA extracted from blood followed the Biological Fluids and Cells protocol and DNA extracted from placenta followed the Solid Tissue protocol. DNA abundance and quality were determined after extraction using a Nanodrop ND‐1000 spectrophotometer (Thermo Fisher Scientific, Waltham, Massachusetts, USA). Absorbance ratios (A260/A280 and A60/A230) were above the recommended 1.8. DNA from each sample was further quantified on a Qubit® Fluorometer using Qubit® dsDNA HS Assay kit (Cat. No. Q32854, Thermo Fisher Scientific, Waltham, Massachusetts, USA).

### 2.3 Methyl‐Seq library preparation

Methyl‐Seq was performed using the SureSelect^XT^ Methyl‐Seq kit (Cat. No. G9651B, Agilent, Santa Clara, California, USA) according to the manufacturer's protocol (SureSelect ^XT^ Methyl‐Seq Target Enrichment System for Illumina Multiplexed Sequencing protocol, version C.0, January 2015); this covers over 3.7 million individual CpG dinucleotide sequences covering CpG islands, CpG island shores, CpG island shelves, under‐methylated regions, promoters, enhancers, transcription factors, CTCF‐binding sites, DNase 1 hypersensitive sites, and DMRs. Three micrograms of DNA from each sample was initially sheared by Covaris sonication to 150–200 bp in size and used to prepare genomic DNA libraries with the SureSelect^XT^ Methyl‐Seq Library Prep kit. After hybridization with the SureSelect^XT^ Methyl‐Seq capture library, targeted regions were isolated using complementary RNA baits. Isolated targets were bisulfite converted using the EZ DNA Methylation‐Gold^TM^ (Cat. No. D5005, Zymo Research) which converts unmethylated cytosines to Uracil, while methylated cytosines are unaltered. Subsequent PCR amplification creates an unmethylated CG→TA transition at unmethylated positions. Each sequence‐modified, target‐enriched library preparation was attached to a readable index (short DNA identifying code) by PCR. Libraries were quantified on a Bioanalyzer 2100 (Agilent, Santa Clara, California, USA) using the Agilent High Sensitivity DNA kit (Cat. No. 507–4626, Agilent, Santa Clara, California, USA) or using a 2200 TapeStation (Agilent, Santa Clara, California, USA) with High Sensitivity DNA ScreenTapes (Cat. No. 5067–5593, Agilent, Santa Clara, California, USA). Equimolar indexed libraries were multiplexed (in groups of 6) to a final concentration of 4 nM in 20 μL of nuclease‐free dH_2_O or 10 mM of Tris–Cl, pH 8.5 (Buffer EB, Cat. No. 19086, Qiagen) and run on a single flow cell on an Ilumina NextSeq 500 according to the manufacturer's instructions using a 2x75 bp paired end read kit giving a total read length of 150 (TG NextSeq® 500 kit High Output Kit v2, Cat. No. TG‐160–2002, Illumina). To overcome color imbalance inherent to low complexity in a bisulfite‐converted genome, 10% of phiX genome was spiked into the reaction. Q30 scores of bases from NextSeq runs were within the threshold recommended by the manufacturer and depth of coverage was approximately 40x.

### 2.4 DNA data mapping

FastQC v0.11.4^28^ (http://www.bioinformatics.babraham.ac.uk/projects/fastqc/) was used to visualize the sequencing quality of the raw reads which were then trimmed using Trim Galore! v0.4.0^29^ (http://www.bioinformatics.babraham.ac.uk/projects/trim_galore/). This removes low quality bases (Qscore <20) starting from the 3’ end of the read. After trimming, short reads are removed (<20 bases). Figure S1 shows a typical example. The Bismark package uses Bowtie 2 alignment software v2.2.6^30^, ^31^ to align sequences to the reference genome (GRCh38/hg19 assemblies) and then, methylation data were extracted employing default settings.^31^ Alignment mapping efficiency was in the region of 80% across all samples and is illustrated by Figure S2. The bisulfite error rate, estimated from the methylation status at cytosines outside a CpG context was in the region of 1.0% (Figure S3). Duplicated reads (removed using Bismark) were in the region of 20%. At each cytosine site, the methylation level was calculated as the ratio of the count of “C” (or the number of sequencing reads with methylated cytosine) to the count of “C” plus “T” (or the total number of reads covering that site). M‐bias plots were generated after methylation data were extracted with Bismark to yield the percentage methylation across all reads in order to identify any bias (e.g., bias at the end of the read due to drop in quality) arising from the position in the read of the cytosine residue being called. Figure S4 illustrates an example (for infant bloods) of an M‐bias plot illustrating the reduction of call quality at the 5’ and 3’ ends of the paired reads. This provides a guide to the extent of necessary sequence trimming (typically four bases removed from the 5’ end and one from the 3’ end). Methylation information was then re‐extracted and the output was processed and converted to a bedgraph.

### 2.5 Methylation data analysis

The resulting bed files from Bismark were used for further statistical analysis. Three comparison groups based on different growth criteria were examined: (a) high versus low birthweight babies sampled at birth for placenta and cord blood, (b) high versus low birthweight groups sampled at 12 m for infant blood, and (c) high versus low length‐for‐age based on change in Z score (LAZ) between birth and 12 months sampled at 12 months for infant blood. Differential methylation between groups was examined using BiSeq.^31^, ^32^ Only CpGs covered by at least 10 reads were included in the analysis.

### 2.6 Detection of DMRs

Analysis was performed using R v 3.2.2^33^ and BiSeq version 1.18.0 (32, see review 34). BiSeq is designed specifically for targeted bisulfite sequencing data and includes features such as limiting high coverage, removing low coverage, spatial correlation, a multiple testing correction, visualization, and genomic annotation. DMRs were detected by comparing birthweight categories (high vs. low) or length‐for‐age (LAZ) scores (high vs. low) and incorporated sex as a covariate. Briefly, sequences were grouped into clusters of adjacent CpG sites. CpG methylation often occurs in clusters and spatial correlation is a key characteristic of DNA methylation. As methylation is conserved across short distances, identification of these related regions reduces data dimensions and also increases detection power by borrowing nearby CpG information. BiSeq CpG clusters were defined as CpG sites covered in at least 25% of samples (defined as frequently covered CpG sites) with a maximum distance of 100 bp between CpG sites within a cluster and with clusters containing at least 5 of these CpGs. To mitigate sequence overrepresentation distorting the data, sequences with greater than 90% of maximum coverage were removed. The methylation data were smoothed within CpG clusters using the smoothing algorithm (“predictMeth”). This estimates the true methylation level of each site in each sample. The methylation data were tested for both the test groups and resampled datasets under the null hypothesis that differences in methylation are random. The data from both were modeled by beta regression, with the group as the independent variable and the methylation probability as the dependent variable. A Wald test was used to confirm the parameters used in the beta regression could be included in the model and associated *p*‐values were transformed into Z scores to allow DMRs to be detected.^31^ To account for multiple testing errors (multiple testing correction using the Benjamini–Hochberg method),^35^ a two‐step hierarchical procedure was employed. This first tests clusters, then individual CpG sites within those clusters. The two‐step approach avoids loss of power by first testing at the cluster level and then, the CpG in the cluster that showed a change in methylation and hence the number of CpGs needing correction is greatly reduced. A variogram was created under the null hypothesis, which estimates the correlation in methylation between two CpG sites within a cluster. This was plotted and smoothed, with a sill of 1 for all our tests and was combined with the Z scores of the test results of interest to estimate the correlation of Z scores between two locations in a cluster. Clusters without differentially methylated CpG sites were removed (FDR >= 0.1), before the remaining clusters were trimmed to indicate individual significant CpG sites (FDR <= 0.05). PCA analysis of methylation patterns determined from different DNA sequence runs did not reveal any batch effects.

### 2.7 Pyrosequencing

Validation of differentially methylated cytosines as detected by Methyl‐Seq was performed by bisulfite pyrosequencing on the *ZFHX3* gene. Initially, PCR primers were designed using the Pyromark assay design SW 2.0 (Cat. No.9019077, Qiagen, USA) and were supplied by Sigma‐Aldrich, UK. One of the primers was biotinylated and purified by HPLC. The primers were; *ZFHX3*: forward PCR primer GTTTTAATTTGATTGGGGGGAAAG, reverse PCR primer CCTTTAACAAACTAACCTCCTAACA, and forward biotinylated sequencing primer TTTTTTTAAATGTAGATTTGAATT.

PCR amplification was performed with 10 ng of bisulfite converted DNA using EpiTaq HS (Cat. No. R110A, TaKaRa Bio Inc, Japan). PCR was set up according to the manufacturers’ instructions but the concentration of MgCl_2_ varied between 15 and 25 mM dependant on the primer set. Both methylated and unmethylated controls from the EpiTect PCR control DNA kit (Cat. No. 59695, Qiagen, USA) were run alongside. Thermal cycling conditions were performed using a touchdown program with an annealing temperature range of 53°C–62°C and cycle number range of 25–35, dependant on primer set. The PCR products were electrophoresed on a 3% of agarose gel to check for product specificity. Pyrosequencing was then performed on the PyroMark Q24 Vacuum Workstation (Qiagen, USA) as described in the manufacturer's instructions. PyroMark CpG software Design 2.0 (Cat. No. 9019067, Qiagen, USA) was used in this assay and primers with the best quality score were selected. Bisulfite conversion was shown to be efficient for all samples as the fluorescence signal by cytosine in a non‐CpG context was ≤1% of the signal produced by thymine.

### 2.8 Cellular heterogeneity assessment between sample groups

For cord blood, cell composition was compared between low and high birthweight groups using overlaps with a cord blood cell type‐specific reference panel of 215,000 CpGs derived from the Illumina EPIC 850 k array (ref: https://www.ncbi.nlm.nih.gov/pmc/articles/PMC6284779/). The reference panel set of CpG loci was used to find overlaps with the processed Methyl‐Seq capture dataset. Co‐methylation patterns extend up to several 100 base pairs across CpG clusters.^36^, ^37^ In order to obtain enough coverage for the regions covered by the EPIC reference set, we used intervals of 200 bases centered around the locations of the EPIC reference CpG set (updated in Human Genome––HG19). This yielded 14993 regions each containing CpG loci as present in the processed methyl capture sequence dataset. Methylation calls were extracted from the processed sequence data as described above and the mean values in these regions were used to generate PCA plots and heatmaps to calculate the correlation values between experimental groups (using Pearson correlation). In the absence of a 12‐month blood reference panel, the adult blood reference panel based on the Illumina Infinium HM450 k and EPIC 850 K methylation chips^38^, ^39^ was used and processed in the same way for coverage across the Methyl‐seq capture dataset. An interval of 200 bases yielded 33 regions each containing CpG loci (providing coverage for CD4 and CD8 lymphocytes, NK cells, neutrophils, B‐cells, and monocytes) that are present in the processed methyl capture sequence dataset to compare the 12‐month groups.

### 2.9 CpG and gene annotation

Ensembl was used to annotate differentially methylated CpGs (based on hg38 version GRCh38 human genome build) to determine their location with respect to regulatory features. Ontologies, mutational and functional data of those genes associated with significant differentially methylated CpGs were determined using the U.S. National Center for Biotechnology Information (NCBI; Bethesda, MD, USA; http://www.ncbi.nlm.nih.gov/) Gene, Online Mendelian Inheritance in Man (OMIM), PubMed databases and the Database for Annotation, Visualization, and Integrated Discovery v6.7 (DAVID ‐ http://david.abcc.ncifcrf.gov/).^40^ Disease associations were determined by interrogating the Genetic Association Database (GAD) for complex diseases and the EBI GWAS Catalogue. PANTHER v14.0 (http://www.pantherdb.org) ^41^, ^42^ was used to provide an overview of gene ontology (GO Terms) defining protein classes, cellular components, biological procesess, and molecular functions of genes implicated by methylation marks.

## 3 RESULTS

### 3.1 Quality Triage of Sample Cohorts

All samples underwent assessment to exclude maternal contamination and poor‐quality samples. Maternal blood contamination of cord blood (for both sexes) was assessed using marker CpGs that are only methylated in adult blood DNA, and maternal contamination of placenta trophoblast samples from males was also flagged by examining the levels of Y DNA methylation dilution^43^; see Figure S5a,b Poor quality and/or obvious outlier samples were identified by plotting a heatmap of the methylation data for each experimental group (see example of the methylation data from the birthweight cohort at 12 months of age in Figure S6). The major component of variation was sex. Principal Component Analysis (PCA––done with and without inclusion of the sex chromosomes) matrices were also applied to a list of available variable information for the subjects contributing to each cohort and tissue sample (see Table S1) to determine whether they had a significant effect on the variation in the data. Examples for sex (male/female), birthweight category (high/low), and season (dry/wet) are illustrated in Figures S7a,b,c; only sex contributed significantly to variation in the data. Sample sets emerging from these analyses were re‐analyzed with sex as a covariate.

### 3.2 Assessment of Confounding Cellular Heterogeneity

There is no available cell‐type‐specific reference set for cord and adult blood to assess cell composition changes based on DMRs detected by Methyl‐Seq data. Potentially confounding differences in cell‐type composition between cord blood groupings (high and low birthweight) and infant blood groupings (high and low birthweight and length for age) were, therefore, assessed using DMR regions within the capture DNA sequence dataset that overlap within a 200 base‐pair interval with the EPIC 850 k cord blood and Infinium HM450 k adult blood cell‐type‐specific CpG panels (see methods). The adult overlaps were used for the 12‐month infant blood data in the absence of an age‐related reference panel for this time‐point. The heatmap and PCA plots are shown in Figures S8 and S9. For both cord and infant blood data, the heatmaps indicate high correlation between samples and no clear clustering according to comparison groups. PCA analysis indicates inter‐individual differences in cellular composition. For the small number of probes from the adult blood reference panel present within the Methyl‐Seq capture DNA sequence dataset, the analysis shows separation of individual samples into two groups; this may reflect the small number of probes available and their disproportionate weighting or variation in the rate of loss of nucleated erythrocytes between individuals. However, in all three PCA plots the variation between samples captured in PC1 and PC2 is distributed fairly uniformly across both experimental groups (high or low birthweight or tall or short height for age) indicating little difference in cellular composition between comparison groups to confound the determination of differential methylation values at the same time‐point.

### 3.3 Differentially Methylated Loci Identified Through BiSeq Analysis

The total number of significant differentially methylated regions (DMRs) and the direction of median methylation change identified for each comparison group (placenta‐birthweight, cord blood‐birthweight, infant blood‐birthweight at 12 months, and infant blood‐LAZ) by BiSeq analysis and the total number of significant CpGs they contain is shown in Table S2a–d and summarized in Table 3; 194 CpG loci in total. Each significantly differentially methylated CpG in each DMR was examined for the presence of single‐nucleotide polymorphism (SNP) directly in the CpG; these are shown in Table S2a–D. Apart from three SNP‐containing CpGs, all the MAFs (minor allele frequencies) were <0.01. For the CpGs associated with implicated genes *RPS6KA2*, *PRSS3*, and *GAR1*, the MAFs were <0.03, <0.11, and <0.02, respectively. These MAFs are at a level that would not significantly alter the estimation of methylation differences between test groups.

**TABLE 3:** Summary of Numbers of DMRs, CpG Loci, and Implicated Genes

Cohorts	Total Number of DMRs	Direction of Median Methylation Change for DMRs Relative to High Groupings for Birthweight and LAZ		Total Number of CpG sites in DMRs and implicated genes
		+ve	‐ve	
Placenta BW	4	2	2	4 (4)
Cord blood BW	68	25	43	88 (78)
Infant blood (12 m) BW	54	29	25	71 (65)
Infant blood (12 m) LAZ	25	13	12	31 (26)

Summary of total number of DMRs, CpG loci, implicated genes (in brackets), and direction of median methylation change identified from the comparisons made at each time‐point between groupings. Key: BW, Birthweight; LAZ, Length for age Z score. Median methylation change is expressed relative to the high birthweight and high length for age groupings.

**FIGURE 1:**
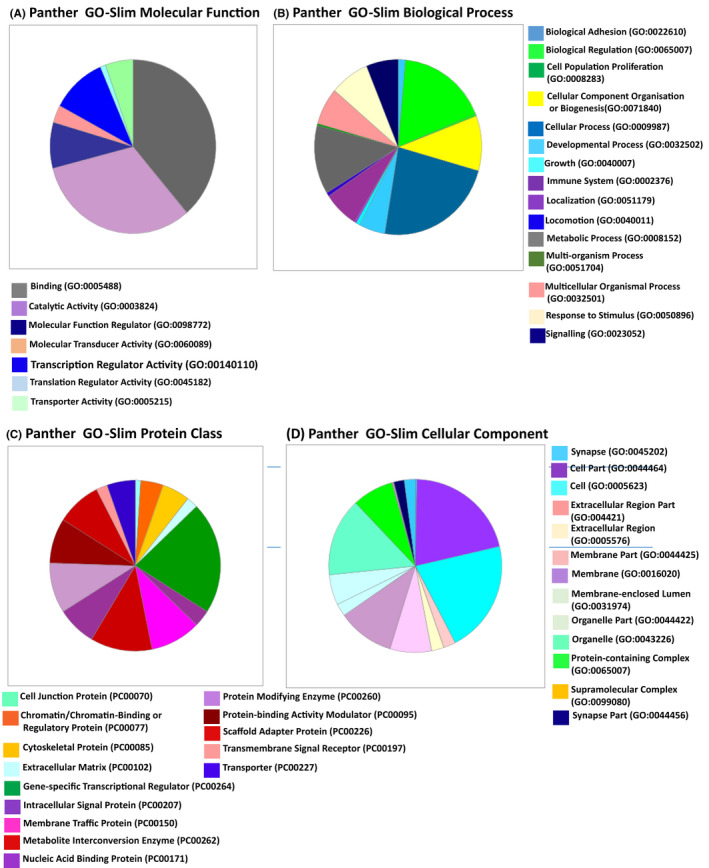
Gene Ontology Analysis of Genes Implicated by Associated Methylation Marks. Panther version 14 was used to provide an overview of the gene ontology characterizing genes implicated by methylation marks. Panther 14.0 identified 161 hits from the uploaded list of 173 genes. A. GO terms for Molecular Function found 93 molecular function hits. B. GO terms for Biological Process found 276 process hits. C. GO terms for Protein Class found 94 class hits. D. GO terms for Cellular Component found 300 cellular component hits. Color coding has been assigned starting at 12 o'clock and working clockwise on the pie chart

The distribution of CpGs between gene regulatory elements, gene bodies, and intergenic regions is shown in Table 4 (see Table S2a–d for full details on all DMRs and CpG locations). Figure 1A–D provides an overview of the gene ontology (determined using PANTHER v14.0 available at http://www.pantherdb.org) characterizing genes implicated by differential methylation marks. The pie charts summarize the distribution of this gene set across GO terms defining molecular functions, biological processes, cellular components, and protein classes. It can be seen that certain GO categories predominate. For example, analysis of molecular function reveals that binding, catalytic activity, molecular function regulator, and transcriptional regulator activity are most prominent. Detailed information on the genes and proteins in each of the GO categories can be obtained by uploading the gene lists to http://www.pantherdb.org from Table S2a–b and interrogating each pie chart sector.

**TABLE 4:** The distribution of methylation marks between regulatory features, gene bodies, and intergenic regions

Genomic Feature	Number of CpGs in Feature	Number of CpGs in Feature	% of Total
	>5% median methylation change	<5% median methylation change	
Promoter	30	44	37.9
Promoter and CTCF‐binding site	9	3	6.2
CTCF‐binding site	11	3	7.2
Transcription Factor‐binding site	4	1	2.5
Enhancer	0	1	0.5
Exon	16	5	10.8
Intron	28	9	19.5
Intergenic	17	13	15.4

Very few of the differentially methylated CpGs found in DMRs identified from the cord blood comparisons are found in the 12‐month infant blood comparisons; (a) of the four closely linked CpGs associated with *TNXB*, one (upstream intergenic) is differentially methylated in the cord blood birthweight group and the remaining three (within intron 1 of the gene) in the 12‐month infant blood birthweight group and (b) the intergenic CpG upstream of the *HLX* gene is differentially methylated in both the 12‐month birthweight and 12‐month length for age groups.

### 3.4 CpG Loci Showing 5% or Greater Methylation Change

**FIGURE 2:**
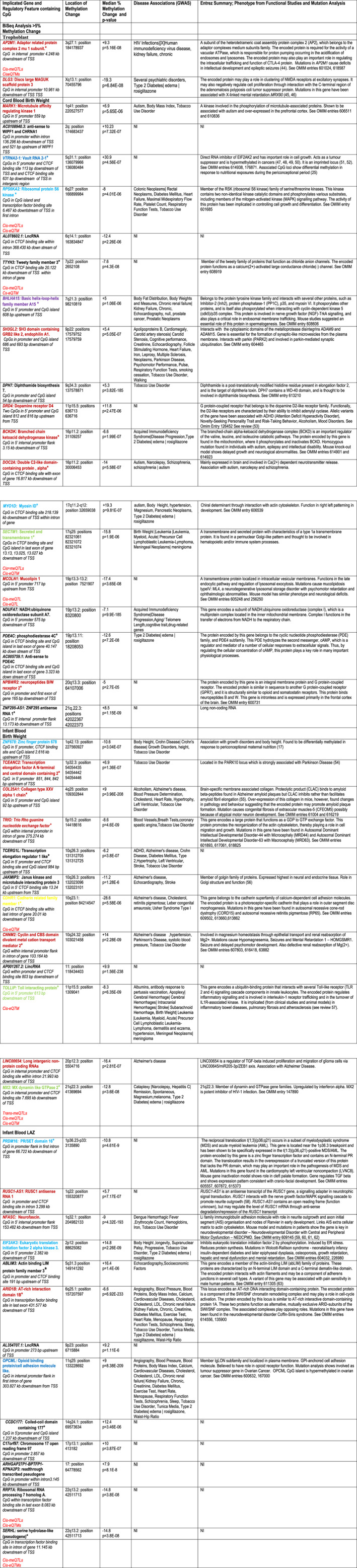
Implicated Genes Associated with Methylation Changes of 5% or Greater in Regulatory Elements. This figure documents those genes where a methylation change of 5% or greater has occurred within a defined regulatory feature. It also provides a summary of function and any disease associations resulting from genome‐wide association studies (GWAS––culled from the GAD and EMBL genetic association catalogue databases), mutation analysis, and functional investigations. The methylation change is expressed relative to the high birthweight and high length for age groups. All mapping of DMRs is based on human genome build hg38 version GRCh38 of the human genome. The positions of CpGs is given in relation to the Transcription Start Site (TSS) of the implicated gene. Also shown highlighted in red in the first column is whether the gene is associated with Trans and/or Cis‐meQTLs and/or Cis‐eQTMs. Where plural is shown, this indicates two or more Trans‐meQTLs, Cis‐metQTLs, or Cis‐eQTMs associated with the gene (information obtained from the BIOS QTL Browser at www.genenetwork.nl/biosqtlbrowser). NI, No Information. Color key of gene disease and functional associations: brown, neurological; purple, fertility; light blue, growth and development; dark blue, oncological; and light green, immunological. The asterisks mark the implicated genes found associated with methylation marks in related studies.^15^, ^86^, ^87^

**FIGURE 3:**
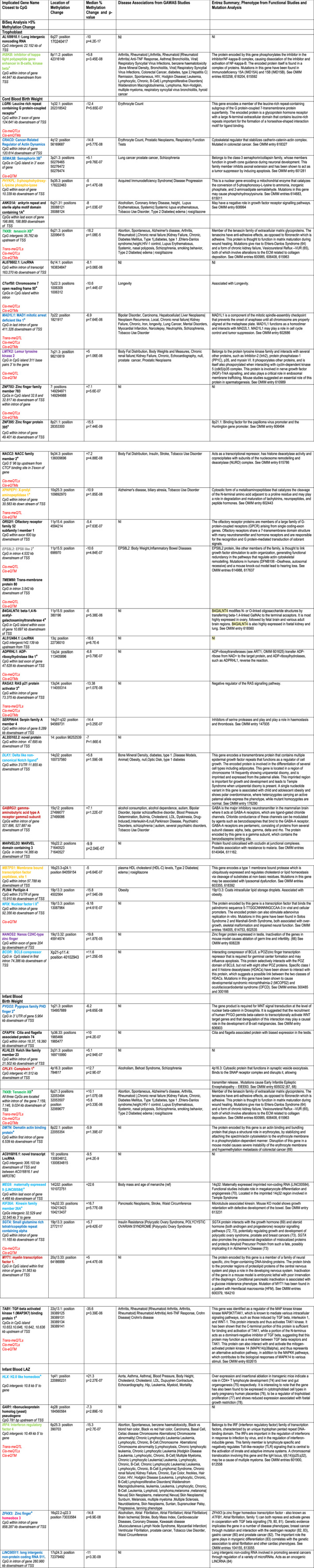
Implicated Genes Associated with Methylation Changes of 5% or Greater in Gene Bodies and Intergenic Regions. This figure documents those genes where a methylation change of 5% or greater has occurred within a gene body or intergenic region. It also provides a summary of function and any disease associations resulting from genome‐wide association studies (GWAS––culled from the GAD and EMBL genetic association catalogue databases), mutation analysis and functional investigations. The methylation change is expressed relative to the high birthweight and high length for age groups. All mapping of DMRs is based on human genome build hg38 version GRCh38 of the human genome. The positions of CpGs is given in relation to the Transcription Start Site (TSS) of the implicated gene. Also shown highlighted in red in the first column is whether the gene is associated with Trans and/or Cis‐meQTLs and/or Cis‐eQTMs. Where plural is shown, this indicates two or more Trans‐meQTLs, Cis‐metQTLs, or Cis‐eQTMs associated with the gene (information obtained from the BIOS QTL Browser at www.genenetwork.nl/biosqtlbrowser). NI, No Information. Color key of gene disease and functional associations: brown, neurological; purple, fertility; light blue, growth and development; dark blue, oncological; light green, immunological; dark green, connective tissue; orange, metabolic; vermillion, cardiovascular; and gray, hearing. The asterisks mark the implicated genes found associated with methylation marks in related studies.^15^, ^86^, ^87^

We have chosen to focus on those marks that show 5% or greater methylation change. Figure 2 summarizes the CpGs that have been located to regulatory features (promoters, CTCF‐binding sites, and transcription factor‐binding sites––56 CpG loci in total) and figure 3 those located to gene bodies (introns and exons) and closely linked intergenic regions (64 CpG loci in total). Figures 2 and 3 also present the locations of CpGs (based on hg38 release 85 from ENSEBL) with respect to the Transcription Start Site (TSS) of genes implicated by location (93 in total), the median *p*‐value for the DMR corrected for multiple testing, direction and change in median methylation value, GWAS disease associations, and a short vignette summarizing any mutational data and functional studies of implicated genes culled from the various databases outlined in the materials and methods. Finally, Figures 2 and 3 flag whether any of the DMR‐associated genes are also subject to Trans or Cis‐meQTLs (genetic variation that influences methylation at CpG sites adjacent to implicated genes) and/or Cis‐eQTMs (variation of methylation that influences expression of an adjacent gene) collated in the Bios QTLBrowser held at www.genenetwork.nl/biosqtlbrowser (85), these are marked in red in the first column). These data were based on the analysis of cohorts from the Dutch population and may only partially reflect genetic variation in the Gambian population with Trans or Cis‐eQTL effects.

**FIGURE 4:**
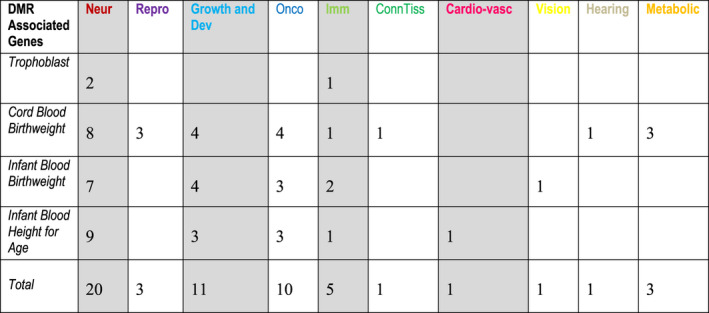
Number of Implicated Genes from Figures 2 and 3 Associated with Different Disease Categories. The color key allows cross‐reference to the gene lists in Figures 2 and 3. Neur, Neurological; Repro, Reproductive; Growth and Dev, Growth and Development; Onco, Oncological; Imm, Immunological; Conn Tiss, Connective Tissue; and Cardio‐vasc, Cardio‐vascular

The implicated gene names in Figures 2 and 3 are color coded according to categories of gene function/disease revealed by functional and/or mutation analysis (see legends) and Figure 4 summarizes the numbers of implicated genes found in associated disease categories bearing the same color coding. It is immediately clear that neurological, growth and development, and oncological disorders are the most prominent among the implicated genes showing 5% or greater methylation change.

### 3.5 Replication of Findings in Other Methylation Studies

Identification of a significant proportion of the same implicated genes reported in related studies provides strong validation of the findings reported here. Highlighted in red bold in Table S2a–d are the DMR‐implicated genes that are also documented in the recent large meta‐analysis of multiple EWAS by the Childhood Epigenetics Consortium examining DNA methylation associated with birthweight.^15^ When all genes (4848, representing about 19% of the estimated 25,000 genes in the human genome) from the consortium study associated with 8170 CpGs significant after FDR correction for multiple testing are screened, 62 DMR implicated genes from the current study show a match (of which 34 show >5% methylation change). If this is restricted to those genes (729; about 2.9% of human genes) associated with 914 CpG loci surviving Bonferroni correction (p < 1.06E‐7), then 11 matches are found (marked with a red asterisk of which 10 show >5% methylation change in the current study). The vast majority of CpGs in the meta‐analysis are located within or closely linked to genes. Thus taking the number of genes in the genome as 25,000, the approximate probability of a match by chance for any given DMR‐implicated gene in the present study is 0.19 (4848/25000) for all genes and 0.029 (729/25000) for those associated with the 914 CpG loci. The probability that these matches have occurred by chance for 62 and 11 genes is 0.19^−62^ and 0.029^−11^, respectively.

Comparisons have been made to two further studies examining the impact of gestational age^86^ (where some of the data are subsumed in the large meta‐analysis mentioned above) and smoking on birthweight^87^; these share, respectively, 53 (marked with a green asterisk in Table S2a–d) and 11 (marked with a blue asterisk) genes associated with differential methylation identified in the current study. These two studies identify a further 22 DMR‐associated genes that overlap with our findings, bringing the replication in other related studies to 84 (48%) of the 173 implicated genes we have documented (The asterisks in figure 2 and 3 mark which of those shared genes show 5% or greater methylation change––in total 49 of the 93 in tables 5 and 6 between the three birthweight‐related studies).

The genes *ZNF678*, *VTRNA2*‐*1*, *SCRIB*, and *TNXB* match those reported in other studies on maternal exposures and differential methylation of associated CpG loci in Gambian infants^22^, ^26^ (marked with black triple asterisks in Table S2a–d) and *SEMA3B*, *ARID1B*, and *HOXA10* from the Cambridge Baby Growth Study^88^ (marked with black double asterisks).

The high degree of replication observed in related studies provides robust validation of the findings reported here. Pyrosequencing analysis of the methylation mark associated with the *ZFHX3* gene was performed to illustrate an example experimental confirmation of methyl‐seq derived methylation data. Table 5 summarizes the data for several individuals selected from the high and low groups for the 12‐month LAZ comparison. The results in Table 5 show good concordance between the two methods in both the quantum and direction of change when compared to the median methylation value change derived from the group comparisons by BiSeq analysis of methyl‐seq data.

**TABLE 5:** Methylation Analysis of the DMR Associated with the *ZFHX3* Gene by Pyrosequencing

*ZFHX3* (% methylation)							
Pyrosequencing				Methyl‐seq			
Sample	High	Sample	Low	Sample	High	Sample	Low
1	20	1	31	1	22	1	40
2	22	2	31	2	22	2	42
3	23	3	31	3	23	3	42
4	23	4	33	4	23	4	41
5	30	5	35	5	24	5	40
		6	35			6	46
Mean:							
23.6 sd+/−3.38		32.6 sd+/−1.79		22.8 sd+/‐ 0.74		41.3 sd+/‐ 1.1	

Individual samples sourced from the 12‐month high and low LAZ (length for age) comparison groups.

Median methyl‐seq determined methylation change between groups = −14.8 referenced to high LAZ value.

This table compares the methylation levels determine for the DMR associated with the *ZFHX3* gene by pyrosequencing and methyl‐seq analysis. Individuals from the high (*n* = 5) and low (*n* = 6) 12‐month length for age comparison groups were selected for analysis. The table shows the mean % methylation values and standard deviation for the two types of analysis. The quantum and direction of change is close to that observed for median methylation change from the comparison of the high and low groups determined by BiSeq analysis of methyl‐seq data.

## 4 DISCUSSION

It has been suggested that epigenetic changes may be involved in the mechanism of reprogramming induced by under‐nutrition, infection, and adverse environmental exposures, although, it is not clear whether these are primary or secondary events in the chain of causality. This paper has used extremes of variation in birthweight and subsequent gains in length to examine associated methylation changes in DNA from trophoblast and cord blood DNA from small and large babies, and blood DNA from 12‐month old infants analyzed both according to their size at birth and their change in length from birth to 12 months (LAZ).

The methylation marks found at birth and those at 12 months in relation to birthweight show little longitudinal persistence (see figures 2 and 3 and Table S2a–d). This suggests ongoing epigenetic adjustments, significant changes in blood cellular composition (such as the loss of nucleated erythrocytes^89^ or both in the critical postnatal growth period, and the subsequent infancy‐childhood growth transition (ICT).^90^ Nonetheless, what does persist at 12 months is different, almost completely non‐overlapping methylation patterns (not confounded by cellular composition differences) between the high and low birthweight comparison groups and the comparison groups showing rapid or slow postnatal height gain. These two distinct methylation patterns may reflect different interactions with nutritional, infectious, and other environmental exposures during the postnatal growth phase potentially associated with negative or positive growth trajectories or a combination of both. Thus, any continued challenges (such as those provoked by under nutrition and infection) to homeostasis during the development period may trigger epigenetic programming and shift the timing and duration of these periods of growth. The study reported by Bernstein et al.^91^ has revealed an accelerated transition to a childhood pattern of growth in Gambian compared to UK infants. A later transition, observed in U.K. infants, extends the high growth rate experienced during the infancy stage. This is reduced in Gambian infants, potentially impacting on growth outcomes in childhood while diverting energy into other processes critical for responses to acute infectious challenges; later developmental stages in this population offer an extended window for catch‐up growth.

Over half (54.3%) of the identified methylation marks are located in gene regulatory elements, 30.3% in gene bodies, and the remaining 15.4% in intergenic regions closely linked to implicated genes. Alteration of gene activity by methylation of implicated genes may occur by impacting the functionality of cis‐transcriptional regulatory elements or changing chromatin conformation and accessibility to the transcriptional machinery. Several of the methylation marks are found in the binding site (an estimated 326,000 in the human genome) for the multifunctional CTCF zinc finger protein. This protein plays a key regulatory role through a number of varied functions that include influencing chromatin architecture (binding at chromatin domain boundaries and the formation of chromatin loops), binding to promoters, enhancers and within gene bodies, and recruitment of transcription factors. The protein can also act as an insulator, blocking long‐range promoter‐enhancer interactions for review see (92). Of particular relevance is the observation that methylation at CTCF‐binding sites in imprinted regions can disrupt the binding of the CTCF protein and its insulator activity^93^ and, more generally, at many other methylated sites outside imprinted regions.^94^ From the annotation associated with each of the CpG loci covered by this methyl‐seq capture set, almost all the methylation marks described in this study are in regions containing DNAse 1 hypersensitive Sites (DHS–markers of DNA regulatory regions and transcriptionally active open chromatin) described by the ENCODE (Encyclopedia of DNA Elements^95^ project. The ENCODE project has shown that a small proportion (~ 5%) of DHSs are found in TSS (Transcription Start Site) regions, that most are located in introns and intergenic DNA and that there is cell‐type specificity in the distribution of DHSs. This indicates that the majority of methylation marks reported in our study are potentially in areas of remodeled open chromatin associated with transcriptional activity and may influence target gene activity possibly by altering chromatin architecture. Figure 2 and 3 also indicate that a number of the DMR‐associated genes showing 5% or greater methylation change are subject to trans and/or cis genetic variation (Trans‐meQTL and Cis‐meQTL) that impacts the level of methylation of closely linked CpG loci; in some cases these methylation changes affect gene expression (Cis‐eQTM). One consequence of this polymorphism in the genetic modulation of methylation marks is that it is likely to lead to a diversity of methylation responses to environmental exposures in different populations. Thus interaction between environmental exposures, genetic background and modulation of methylation patterns will have to be assessed for each study population.

Distribution of implicated genes across GO term categories demonstrates that they encompass biochemical and biological functions that include signaling or interaction with signaling pathways; interacting with or acting as receptors; constituents of or interacting with the extracellular matrix; deposition of connective tissue; structure and function of the actin cytoskeleton; trafficking across cellular membranes; cell cycle control and cellular growth; transcription regulation; and metabolic regulation (see Figure 1). Biological functions revealed by functional studies, animal models, and mutation analysis primarily highlight roles in neurological, growth and developmental, neoplastic, and immunological dysfunction (see figures 2, 3, and 4 and Table S2a–d for details). The precise impact of the methylation changes on the expression of implicated genes, however, is unknown and awaits more detailed functional analysis. Nevertheless, the location of these methylation marks within appropriately positioned regulatory elements and gene bodies or in close intergenic linkage to implicated genes, encourages their consideration as biomarkers associated with and the genetic pathways within which they are active in as potential contributors to variation in prenatal and postnatal growth, subsequent outcomes in later life and as possible intervention targets.

In total, 84 genes implicated by DMRs (shown in red bold and flagged by green and blue asterisks‐see Table S2a–d) are shared with DMR‐associated genes reported in the large array‐based meta‐analysis of multiple EWAS by the Childhood Epigenetics Consortium and two further related studies.^15^, ^86^, ^87^ This demonstrates concordance with a substantial proportion (48%) of the genes documented in the current study and provides robust validation of the BiSeq analysis of methyl‐seq data. Eleven matched genes are associated with CpG loci surviving stringent Bonferroni correction in the Kuppers et al. study^15^ (marked with a red asterisk in Table S2a–d). Differences in genetic background, environmental exposures and nutrition between populations contributing to different studies could lead to methylation changes at different CpG loci but still affect DMRs associated with the same implicated genes. In the case of *MAD1L1* and *NFIX*, differential methylation has been detected at the same Bonferroni significant CpG sites that are reported in the meta‐analysis^15^). *MAD1L1* (a component of the mitotic spindle‐assembly checkpoint) has a role in cell cycle control and tumor suppression and methylation levels have been strongly correlated with hepatocellular carcinoma.^96^ It is also a susceptibility gene for bipolar disorder and schizophrenia with a risk allele linked to reward systems in healthy adults.^97^ *NFIX* is most highly expressed in brain, fat, and prostate, is linked to cancer (DNA hypermethylation associated with lung adenocarcinoma––LUAD),^98^ muscle development and dystrophies.^99^ Interestingly, 19p13 microduplications encompassing *NFIX* are responsible for intellectual disability, short stature, and small head circumference.^100^

Three implicated genes match those flagged by methylation changes found in DNA from babies in the Cambridge Baby Growth Study investigating the effects of maternal gestational diabetes or intrauterine growth retardation.^88^ *ARID1B* and *SEMA3B* are potential tumor suppressor genes. *ARID1B* is a chromatin remodeling factor and individuals with *ARID1B*‐related disorder have many phenotypic features including slow growth.^101^ The third gene is *HOXA10* (homeobox A10), whose expression is downregulated in endometriosis but in late gestation is required for proper placental differentiation and function.^100^, ^102^

Implicated genes *ZNF678* (a zinc finger gene), *VTRNA2*‐*1*, *SCRIB*, *and* *TNXB* have been reported in other Gambian‐based studies investigating periconceptional nutritional exposures associated with differential methylation.^11^, ^22^, ^26^ *VTRNA2a*‐*1* is a noncoding RNA gene that functions as a tumor suppressor^47^, ^48^, ^49^, ^50^ and is an imprinted locus.^51^, ^52^ *SCRIB* (a scaffold protein found at epithelial adherens junctions and neuronal presynaptic compartments) can act as a tumor suppressor gene and has been shown to be mutated in severe neural tube defects (see OMIM entry 607733). *TNXB* is an extracellular matrix glycoprotein thought to function in matrix maturation during wound healing. Different pathogenic alleles give rise to Ehlers–Danlos Syndrome^64^ and a form of chronic kidney failure, Vesicoureteral Reflux –VUR,^65^ both of which involve alterations to collagen deposition in the extracellular matrix.

The genes *DLK1* and *MEG9* (LINC00584––long intergenic noncoding RNA) are worthy of further comment given their location within an important imprinted region on chromosome 14 at 14q32. As revealed by maternal and paternal Uniparental Disomy (UPDm and UDPp, respectively), and genetic and functional studies of individual genes encompassed within the locus (for review see OMIM entries 601038, 60563, 611896, 172690, 613648, and ref (103), the region has a major impact on growth and development. The 14q23 locus is complex with a cluster of maternally and paternally imprinted genes, noncoding snoRNAs (small nucleolar organizer RNA), miRNAs (microRNAs), LncRNAs (long noncoding RNAs), and LINC RNAs under the control of an intergenic differentially methylated region (IG‐DMR).^104^ Three genes (*DLK1*, *RTL1*, and *DIO3*) are all expressed from paternal alleles. *DLK1*, containing six epidermal growth factor repeats, has reduced plasma levels in women bearing small for gestational age babies,^105^ is an inhibitor of adipocyte differentiation^106^ and shows genetic association with age of menarche.^107^, ^108^, ^109^, ^110^ *RTL1* is essential for maintenance of fetal capillaries and potentially involved in formation of the chorioallantoic placenta,^111^ while *DIO3* (Thyroxine Deiodinase Type III) is essential for the maturation and function of the thyroid axis.^112^ A further four genes (*MEG3*, *RTL1as*, *MEG8*, and *MEG9*) are all expressed from maternal alleles. *MEG3* is a LncRNA affecting growth and development in *MEG3* knock‐out mice^113^; *RTL1as* is an antisense transcript to the paternally expressed gene *RTL1* and encodes a number of microRNAs that may regulate the expression of *RTL1* ^105^; *MEG8* is a LncRNA involved in the regulation of trophoblast proliferation and invasion, and implicated in spontaneous early abortion^114^ and *MEG9*, a LINC RNA involved in megakaryocyte differentiation and angiogenesis^70^ shows genetic association with body mass index and age of menarche.^107^

The UPDm (no paternal transcripts: Temple Syndrome) phenotype is characterized by prenataland postnatal growth retardation, neonatal hypotonia, precocious puberty, and facial dysmorphism. The UPDp (no maternal transcripts: Kagami–Ogata Syndrome) phenotype is characterized by severe growth retardation, skeletal abnormalities, facial anomalies, and abdominal muscular defects. Trans‐regulation by maternally expressed small noncoding RNAs from the 14q32 region on the activity of other genes in the genome is likely to contribute to these complex phenotypes.^105^ On the maternal chromosome *DLK1* is silenced. The current study shows a 6% methylation difference of a *DLK1* DMR (higher in high birthweight than low birthweight babies). In contrast, *MEG9* is silenced on the paternal chromosome and shows a 22% methylation difference of a *MEG9* DMR (higher in high birthweight than in low birthweight babies). It is not clear what the impact of these methylation marks is on expression levels as they lie outside the immediate promoter within the gene body. Nevertheless, given that both methylation marks are in DMRs containing DHSs marking potentially open chromatin, it is reasonable to suggest that alteration of the methylation landscape in this region of chromosome 14 could impact chromatin architecture and gene activity with a bearing on growth and development outcomes. It is interesting to note that a study examining the effect of maternal periconceptional micronutrient supplementation of Gambian mothers found increased methylation of a *DLK1*‐associated CpG in cord blood DNA from offspring of mothers who had received the supplements.^24^

A number of limitations should be noted. An accessible tissue such as blood as a proxy for methylation changes in other key target tissues will not capture all the relevant alterations in methylation status. However, there is sufficient concordance between tissues to yield a subset of potentially relevant loci.^115^, ^116^, ^117^ Analysis has been performed with males and females combined; hence sex differences in the methylation patterns have not been determined. The sample size is small, nevertheless, as outlined in the methods, BiSeq is designed for the analysis of targeted methyl sequence data and takes advantage of the conservation of methylation across short distances, co‐assessing methylation changes at several individual cytosine residues within intervals of 100 base pairs. This reduces data dimensions and increases detection power by borrowing nearby CpG information and provides a more detailed and statistically significant evaluation of the methylation status across any given genomic region; this has allowed identification of statistically valid differentially methylated CpGs from this small study. Greater coverage (3.7 million CpGs as compared to the Illumina HM450 k and Epic 850 k chips) of the SureSelect targeted sequencing approach of key gene regulatory elements (adjacent and distant, proximal or distal) to genes they control, offers the opportunity to identify additional methylation marks not necessarily scored by the array‐based platforms.

Studies, such as the one reported here, provide associations and not cause and effect relationships between genes and phenotypes. Mutational evidence is helpful in establishing the likelihood that a gene contributes to a complex phenotype. Identification of methylation marks can be useful in that (a) they might act as biomarkers of early life adverse exposures that impact on early growth and may potentially indicate those individuals with higher future disease risks and (b) potentially flag genes that may be useful intervention targets to ameliorate the consequences of stunting. An integrated large scale analysis of inter‐individual variation of methylation marks in relation to genotype (Trans and Cis‐meQTLs), eQTLs (expression quantitative trait loci including Cis‐eQTMs), disease susceptibility, developmental phenotypes, nutrition, and environmental exposures provides a means of potentially unpicking causal relationships and the relevance of implicated genes. Clearly, the most effective approach to mitigate stunting and associated disease susceptibilities would be to ensure healthy nutrition, adequate sanitation, and living conditions early in the life course.

## CONFLICT OF INTEREST

The authors declare that the research was conducted in the absence of any commercial or financial relationships that could be construed as a potential conflict of interest.

## AUTHOR CONTRIBUTIONS

CRQ developed and managed the experimental procedures and KMH performed them; MS and MG devised the initial analysis pipeline and JB and BS finalized the pipelines and provided the bioinformatic support; CRQ performed the methylation data analysis; CAS and NAA provided supervisory support; AMD and SD managed the collection and extraction of DNA from samples; CRQ and NAA wrote the first draft of the manuscript; RMB, NA, DBD, KKO, AMP, and SEM conceived of and designed the HERO‐G study. All authors contributed to manuscript revision, read, and approved the submitted version.

## Supporting information

Fig S1

Fig S2

Fig S3

Fig S4

Fig S5

Fig S6

Fig S7

Fig S8

Fig S9

Table S1

Table S2
