# Supplementary material for: Identification of methylation changes associated with positive and negative growth deviance in Gambian infants using a targeted methyl sequencing approach of genomic DNA
Source: FASEB Bioadv. 2021 Feb 5;3(4):205–30. doi: 10.1096/fba.2020-00101 (PMC8019263; doi:10.1096/fba.2020-00101)
Supplement: Supplementary file 1 — Fig S1 [file FBA2-3-205-s003.pdf]

Supplementary Figure 1

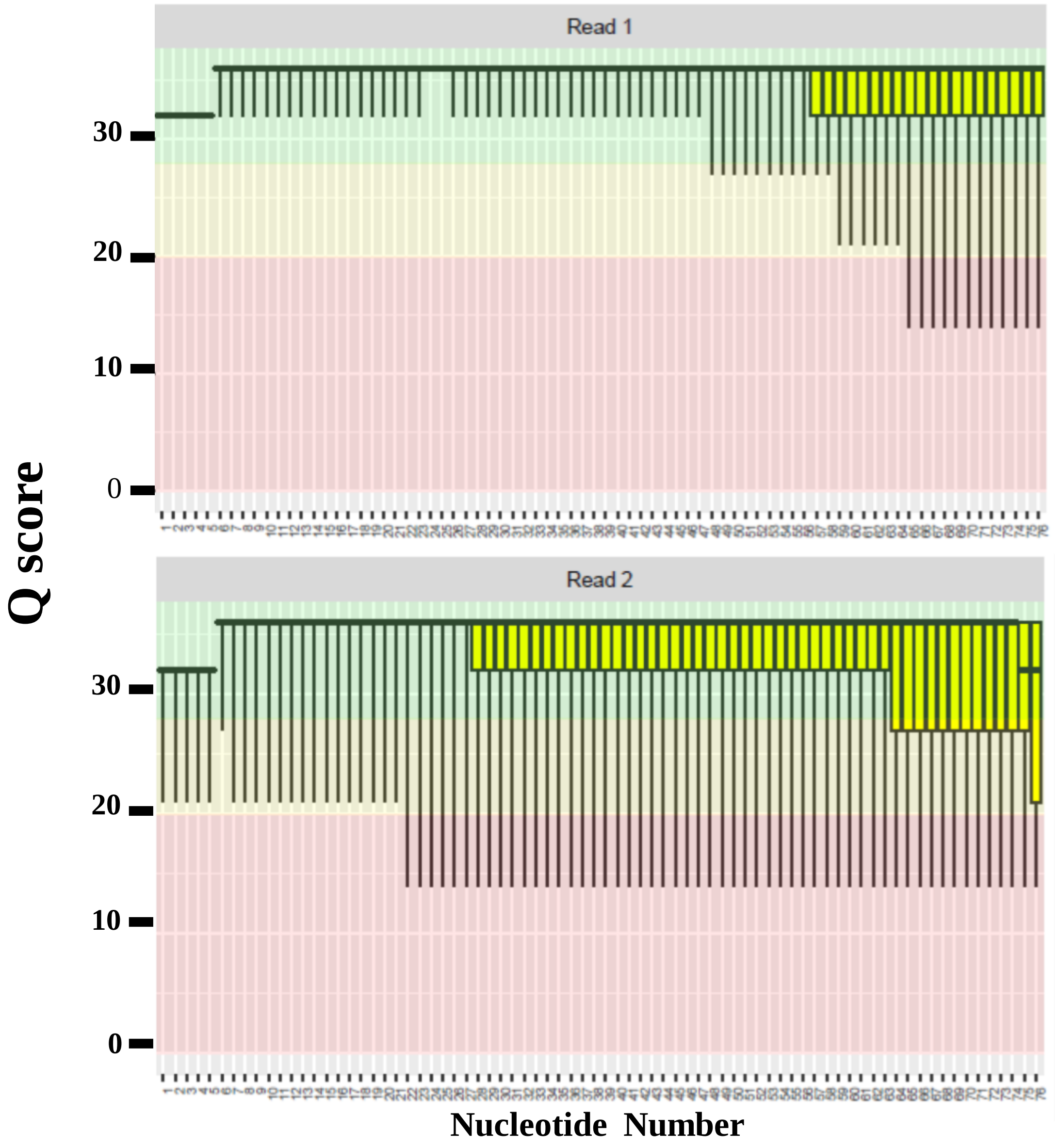

Supplementary figure 1 Illustration of Fast QC of Read Sequence Quality

Example of Fast QC of the sequence quality of reads for one sample. The Q score scale is a Phred Score where Q30 is 1/1000 error and Q20 is 1/100 error. Above 30 Q score is considered good quality; below 20 is considered bad quality. All the bases in this read are considered to be of good quality for read 1, with slight decrease in quality at the ends of the read. Each position of the reads [75bp reads] is represented by a boxplot. The single black lanes represent the outliers of the boxplot, for each position of the reads. The lower limit of the yellow box represent the 25th percentile of the reads Q score at this position. The middle line in the yellow box (last two position of read 2) represent the 50th percentile or median of the reads Q score at this position. The upper limit of the yellow box (75th percentile) is not displayed in this graph due to the scaling, because it is less relevant to read quality than the lower part of the graph. Overall the quality of the bases in both reads is good. R1=forward strand read and R2=reverse strand read.
