# Supplementary material for: Identification of methylation changes associated with positive and negative growth deviance in Gambian infants using a targeted methyl sequencing approach of genomic DNA
Source: FASEB Bioadv. 2021 Feb 5;3(4):205–30. doi: 10.1096/fba.2020-00101 (PMC8019263; doi:10.1096/fba.2020-00101)
Supplement: Supplementary file 2 — Fig S2 [file FBA2-3-205-s010.pdf]

Supplementary Figure 2

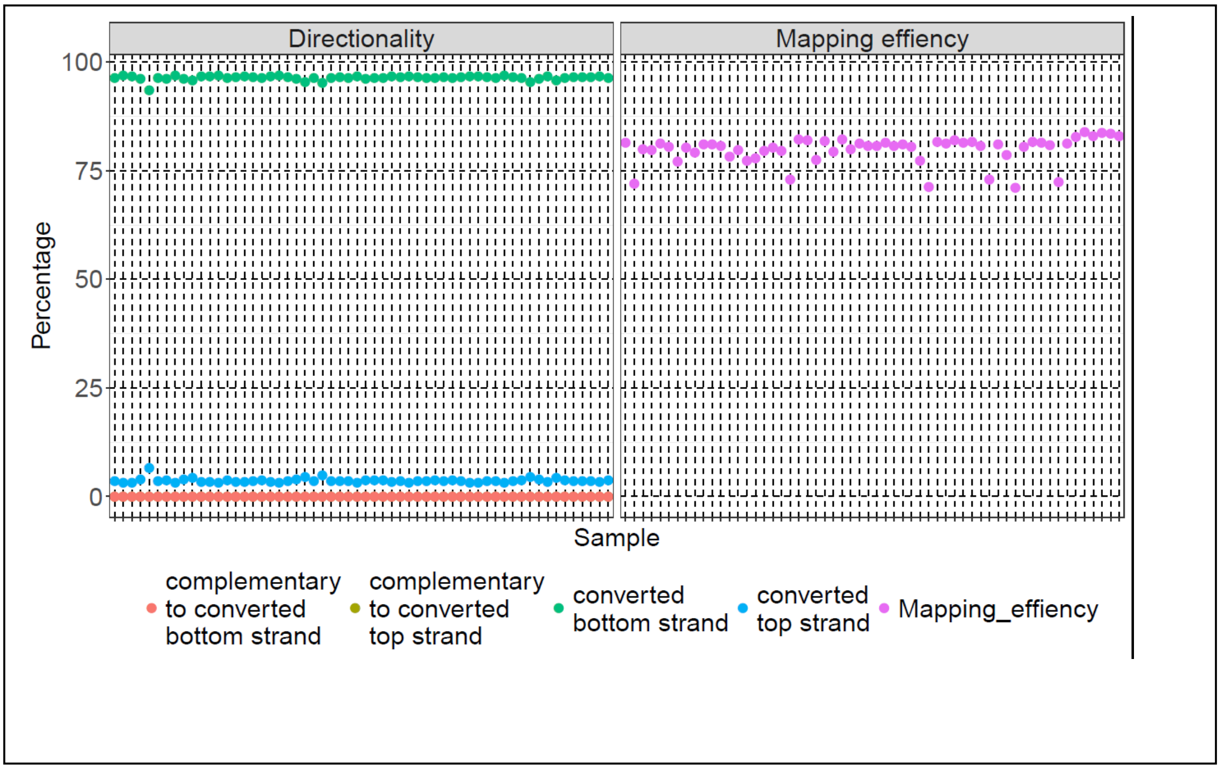

Supplementary figure 2 Mapping QC Efficiency and Directionality

The figure shows an example of mapping QC efficiency and directionality (for infant blood DNA). Each dashed line represents a sample. Bismark by default will try to map against all four possible strands. The majority of the reads mapped to the converted bottom strand which indicated good directionality in the library. The complementary to converted strand (bottom and top) are both at 0 and overlay on top of each other. The mapping efficency reports the percentage of raw reads successfully mapped to the genome.
