# Supplementary material for: Identification of methylation changes associated with positive and negative growth deviance in Gambian infants using a targeted methyl sequencing approach of genomic DNA
Source: FASEB Bioadv. 2021 Feb 5;3(4):205–30. doi: 10.1096/fba.2020-00101 (PMC8019263; doi:10.1096/fba.2020-00101)
Supplement: Supplementary file 3 — Fig S3 [file FBA2-3-205-s005.pdf]

Supplementary Figure 3

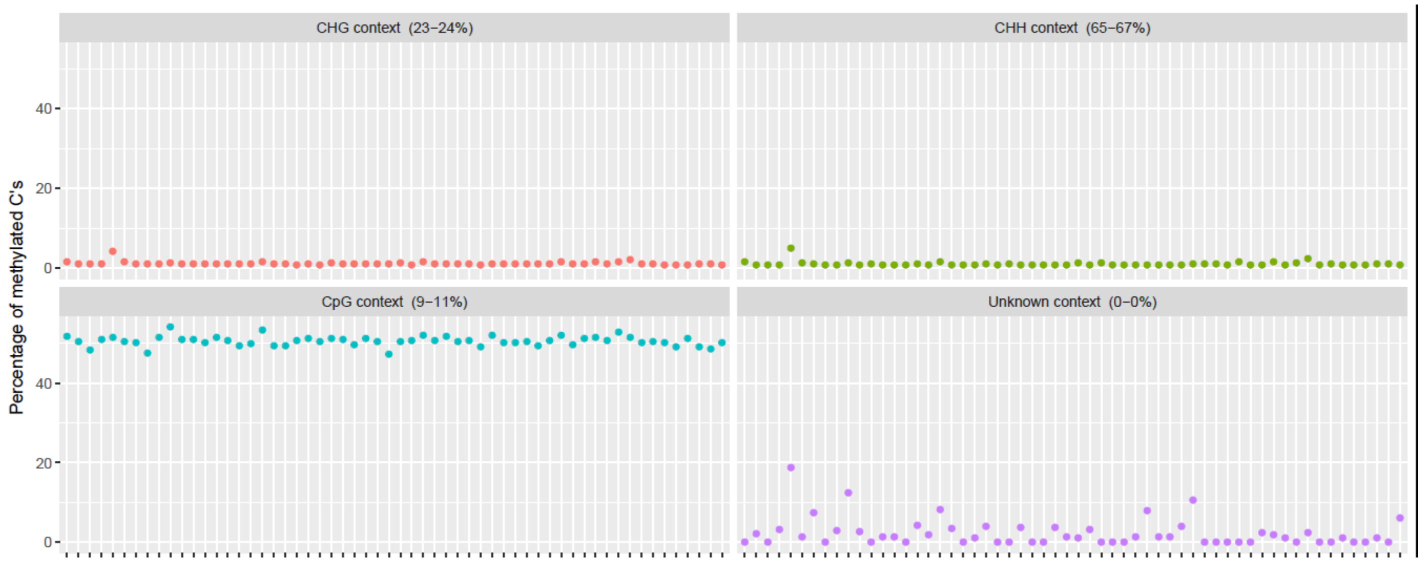

Supplementary figure 3 Plot to Assess the Bisulfite Error Rate

This figure shows illustrates that the bisulfite error rate, estimated from non-CpG context was in the region of 1.0%. The majority of methylated cytosines are found in within a CpG context. Each dot represents a sample.
