# Supplementary material for: Identification of methylation changes associated with positive and negative growth deviance in Gambian infants using a targeted methyl sequencing approach of genomic DNA
Source: FASEB Bioadv. 2021 Feb 5;3(4):205–30. doi: 10.1096/fba.2020-00101 (PMC8019263; doi:10.1096/fba.2020-00101)
Supplement: Supplementary file 4 — Fig S4 [file FBA2-3-205-s007.pdf]

Supplementary Figure 4

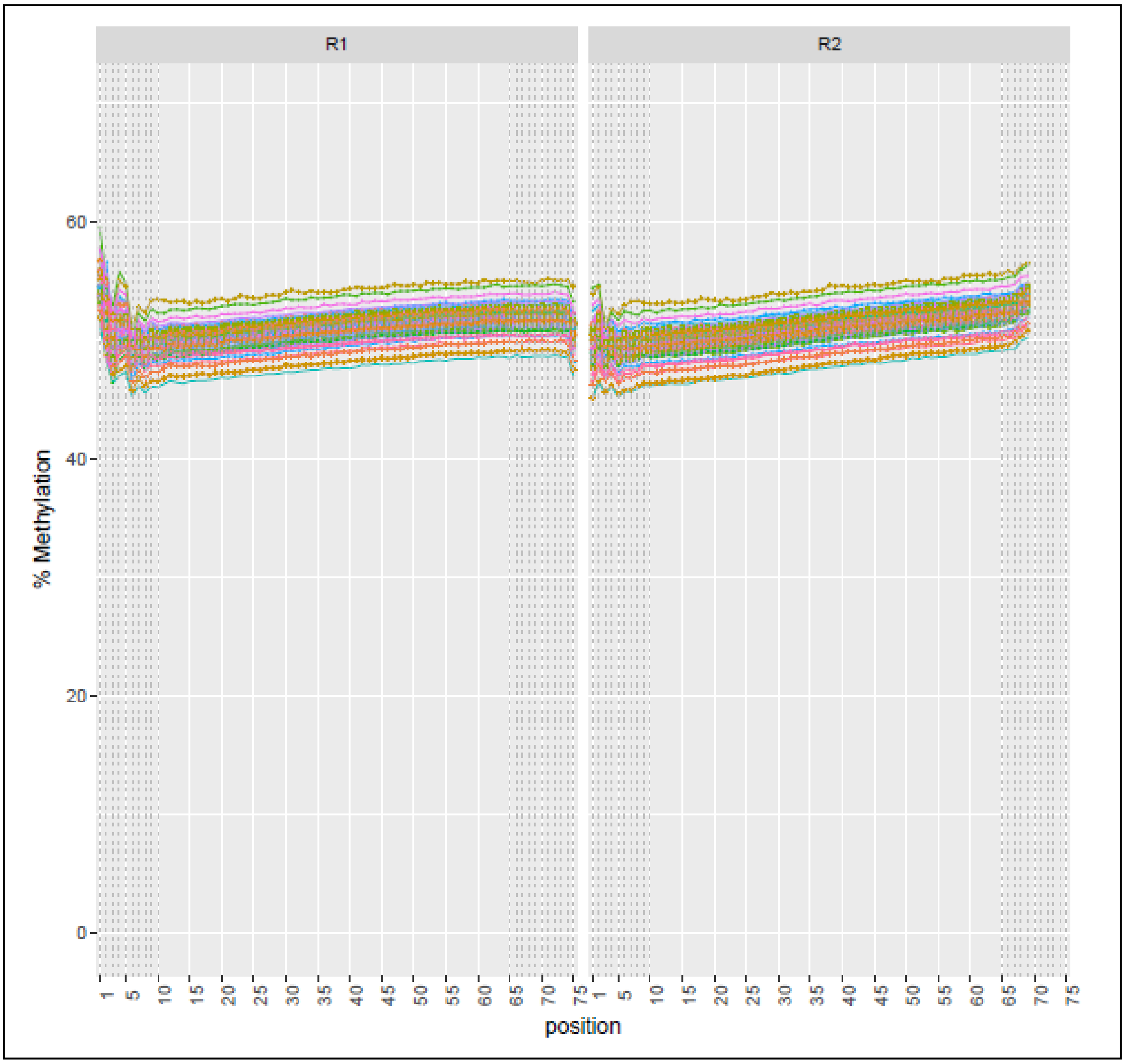

Supplementary figure 4 Example of an Mbias Plot

This figure illustrates an Mbias plot which shows the percentage of methylation extracted using Bismark at each location of the reads. In this case (infant blood samples) there is evidence of some bias at beginning of the reads and at the last base of the read. In general there is very little bias for the majority of the positions. This graph was generated after end bases were removed and methylation re-extracted. Each coloured line represents a sample.
