# Supplementary material for: Identification of methylation changes associated with positive and negative growth deviance in Gambian infants using a targeted methyl sequencing approach of genomic DNA
Source: FASEB Bioadv. 2021 Feb 5;3(4):205–30. doi: 10.1096/fba.2020-00101 (PMC8019263; doi:10.1096/fba.2020-00101)
Supplement: Supplementary file 5 — Fig S5 [file FBA2-3-205-s006.pdf]

Supplementary Figures 5a and b

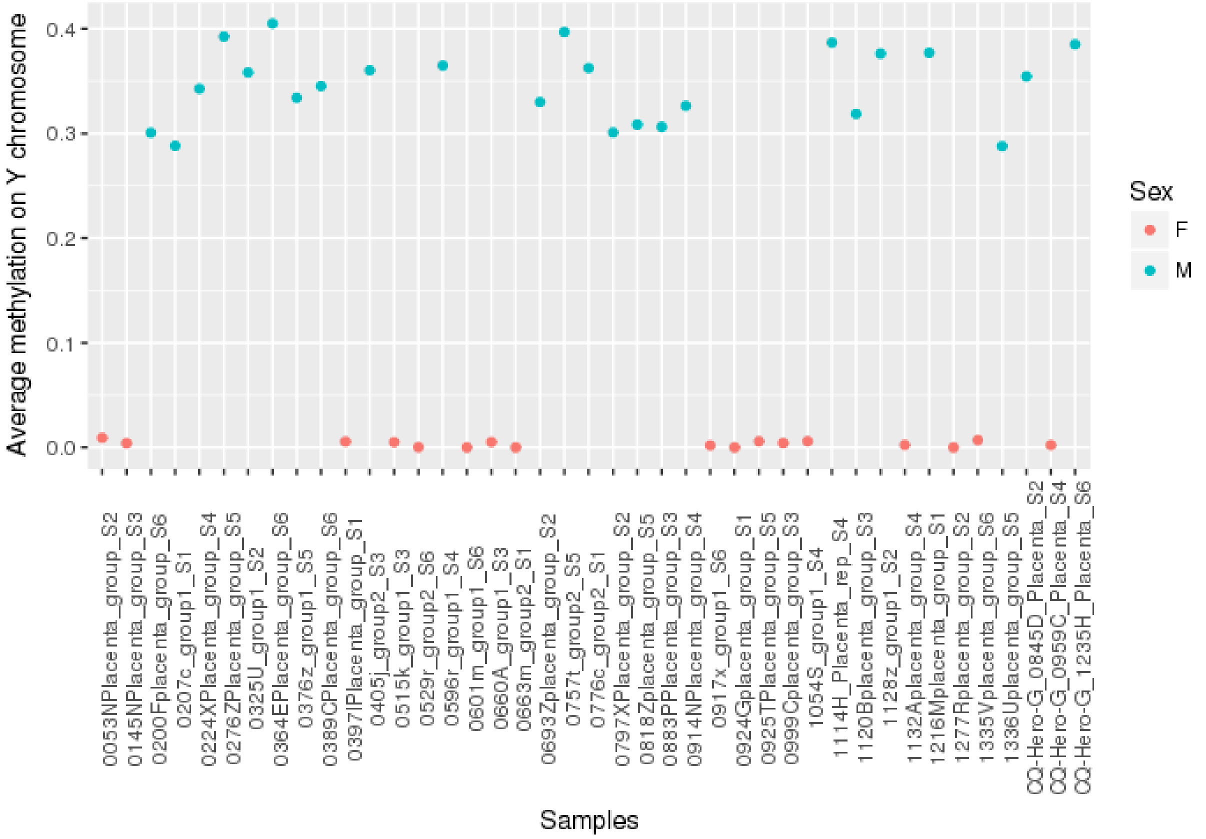

Supplementary Figure 5a Measurement of Y Methylation Levels in Trophoblast Tissue

The figure illustrates that levels of Y methylation are not diluted by maternal blood contamination and only detected in trophoblast material from male births thus excluding cross-contamination between male and female samples. This indicates that there is very little maternal blood contamination in the trophoblast tissue.

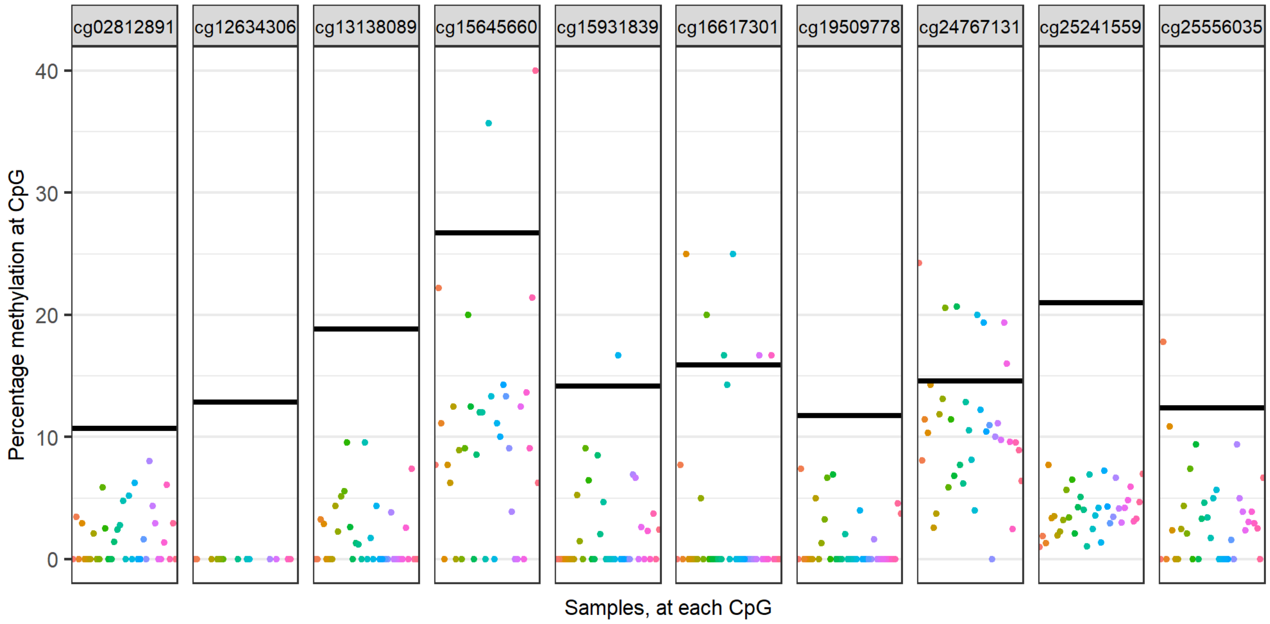

Supplementary Figure 5b Assessment of Maternal Contamination in Cord Blood

This figure shows marker CpGs that are methylated in adult blood (Morin et al. - 41) are nearly all unmethylated in cord blood samples thus excluding maternal contamination. Horizontal lines show threshold values (experimentally determined for each CpG by Morin et al by comparing adult and fetal samples) for distinguishing adult samples from fetal samples by the percentage methylation at each CpG; each dot is a separate sample, coloured sequentially. Morin et al. considered adult contamination to be present in a sample if more than five of these CpGs were above the threshold value; in the cord blood samples, no more than three CpGs were above threshold in any one sample.
