# Supplementary material for: Identification of methylation changes associated with positive and negative growth deviance in Gambian infants using a targeted methyl sequencing approach of genomic DNA
Source: FASEB Bioadv. 2021 Feb 5;3(4):205–30. doi: 10.1096/fba.2020-00101 (PMC8019263; doi:10.1096/fba.2020-00101)
Supplement: Supplementary file 6 — Fig S6 [file FBA2-3-205-s009.pdf]

## Supplementary Figure 6

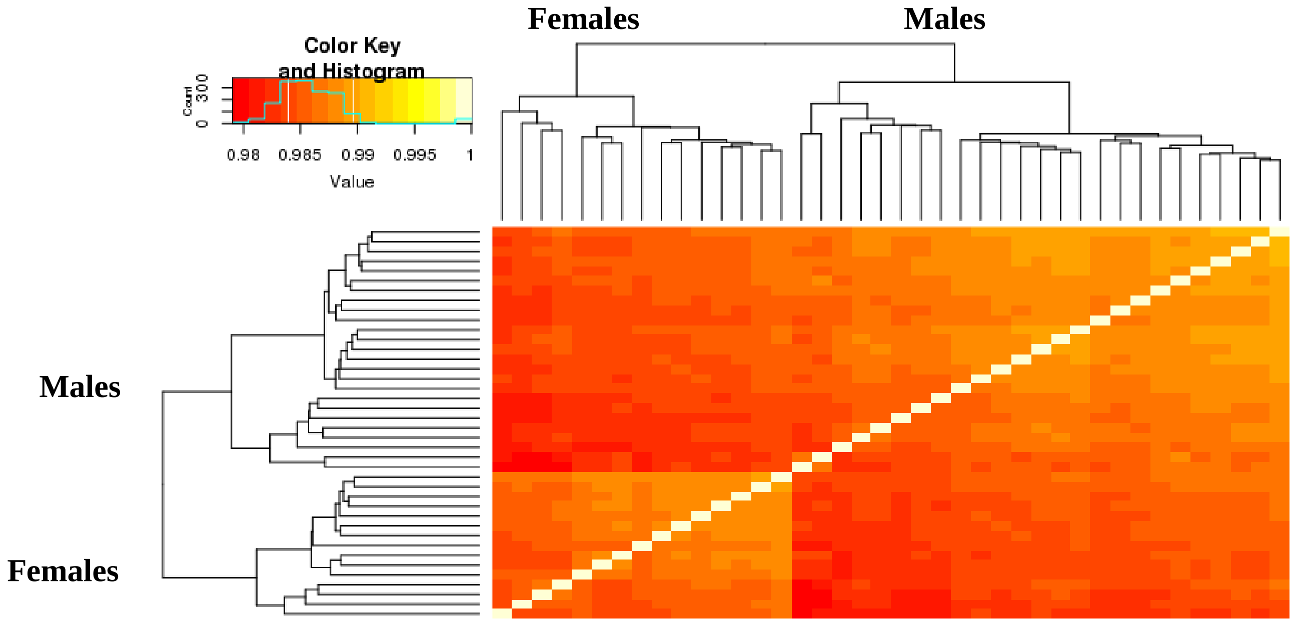


**Spplementary Figure 6 Heat Map to Identify Outlier Samples**

Heatmap plot for infant blood (12m) birthweight cohort after removing poor samples. Hierarchical clustering clearly separates males and females. Each square of the heat map is coloured by the Pearson's R correlation coefficient value according to the colour key and histogram. The white blocks represent the same samples against each other in the matrix yielding a correlation value of 1. Samples are seen separated into groups according to sex.
