# Supplementary material for: Identification of methylation changes associated with positive and negative growth deviance in Gambian infants using a targeted methyl sequencing approach of genomic DNA
Source: FASEB Bioadv. 2021 Feb 5;3(4):205–30. doi: 10.1096/fba.2020-00101 (PMC8019263; doi:10.1096/fba.2020-00101)
Supplement: Supplementary file 7 — Fig S7 [file FBA2-3-205-s002.pdf]

Supplementary Fig 7

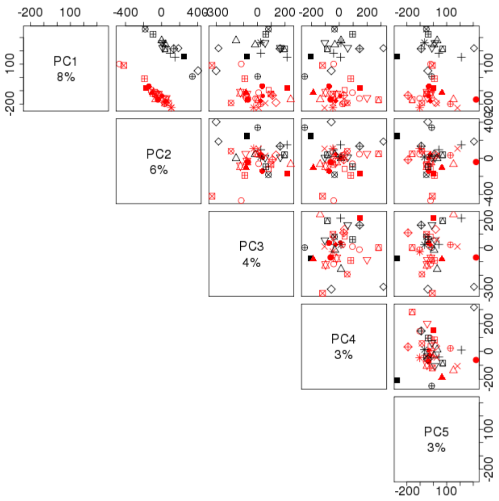

a

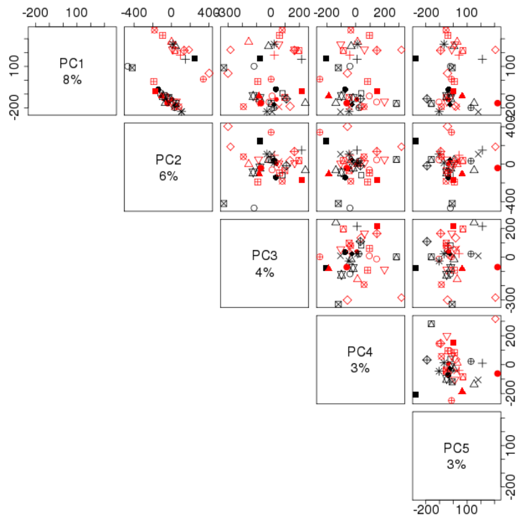

b

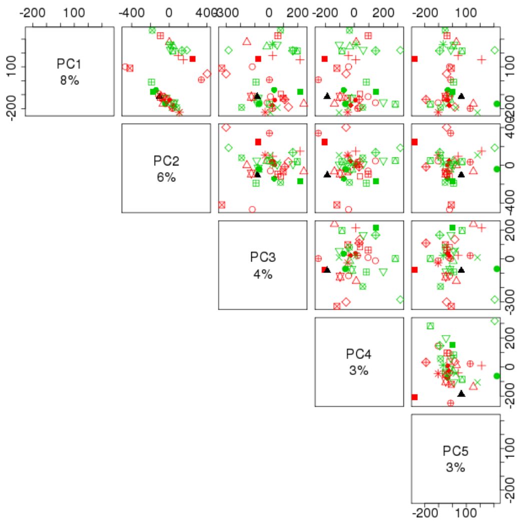

c

Supplementary Fig 7 Principal Component Analysis of 12 Month Infant Blood Methylation

a. PCA plot for infant blood (12m) birthweight cohort shows that principal component 1 versus principal component 2 explains the majority of the variance in the data contributed by sex. (red = male, black = female)
b. PCA plot for infant blood (12m) birthweight cohort for birthweight category, (red = low, black = high) shows that birthweight is not a large contributing factor to the variance.
c. PCA plot for infant blood (12m) birthweight cohort for season of birth shows that season does not have a readily detectable contribution to variance red = wet, green = dry, black = not available.
