# Supplementary material for: Identification of methylation changes associated with positive and negative growth deviance in Gambian infants using a targeted methyl sequencing approach of genomic DNA
Source: FASEB Bioadv. 2021 Feb 5;3(4):205–30. doi: 10.1096/fba.2020-00101 (PMC8019263; doi:10.1096/fba.2020-00101)
Supplement: Supplementary file 8 — Fig S8 [file FBA2-3-205-s001.pdf]

Supplementary Figure 8

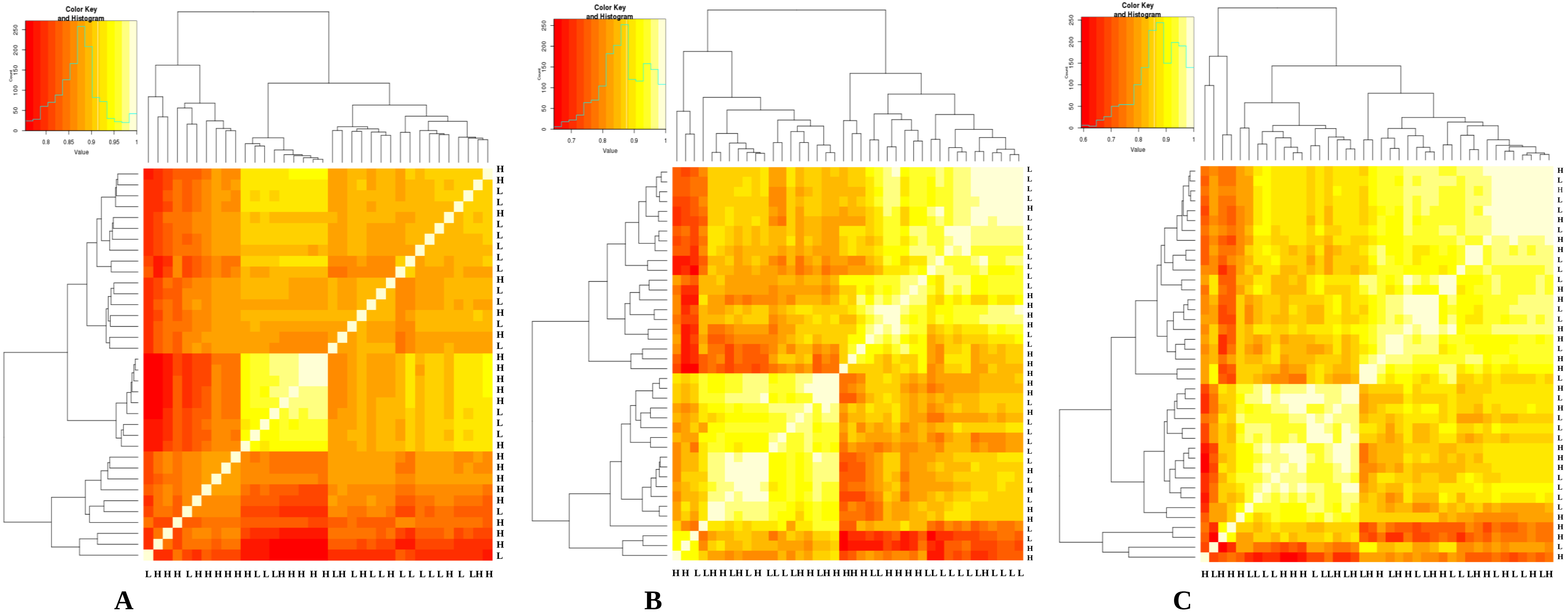

Supplementary Figure 8 Heat Map Plots for Cell Type-Specific CpG Loci

Heatmap plots based on methylation values for cell type specific CpG loci covered by the sequence capture datasets for experimental groups using 200 base pair intervals around the Illumina EPIC 850K cord and Infinium 450K adult cell specific reference CpG panels. Of the 215000 cord blood CpGs, 14993 DMR reference regions were tested. Of the 450 adult blood CpGs, 33 DMR reference regions (representing CD4 and CD8 lymphocytes, NK cells, neutrophils B-cells and monocytes) were tested. Each square of the heat map is coloured by the Pearson's R correlation coefficient value according to the colour key and histogram. The white blocks represent the same samples against each other in the matrix yielding a correlation value of 1. In all three cases, there is a high degree of correlation for most samples and no clear clustering of samples according to experimental group (high or low birthweight or tall or short height for age. A=cord blood high and low birthweight groups tested with EPIC cord blood reference panel, B=12 month infant blood high and low birthweight groups tested with the Infinium 450K adult blood panel , C=12 month infant blood tall and short length for aged groups tested with the Infinium HM450K adult blood reference panel. The adult blood reference panel was used for the 12 month sample sets in the absence of an age-related 12 month specific reference panel. H=high test group and L=low test group.
