# Supplementary material for: Identification of methylation changes associated with positive and negative growth deviance in Gambian infants using a targeted methyl sequencing approach of genomic DNA
Source: FASEB Bioadv. 2021 Feb 5;3(4):205–30. doi: 10.1096/fba.2020-00101 (PMC8019263; doi:10.1096/fba.2020-00101)
Supplement: Supplementary file 9 — Fig S9 [file FBA2-3-205-s008.pdf]

Supplementary Figure 9

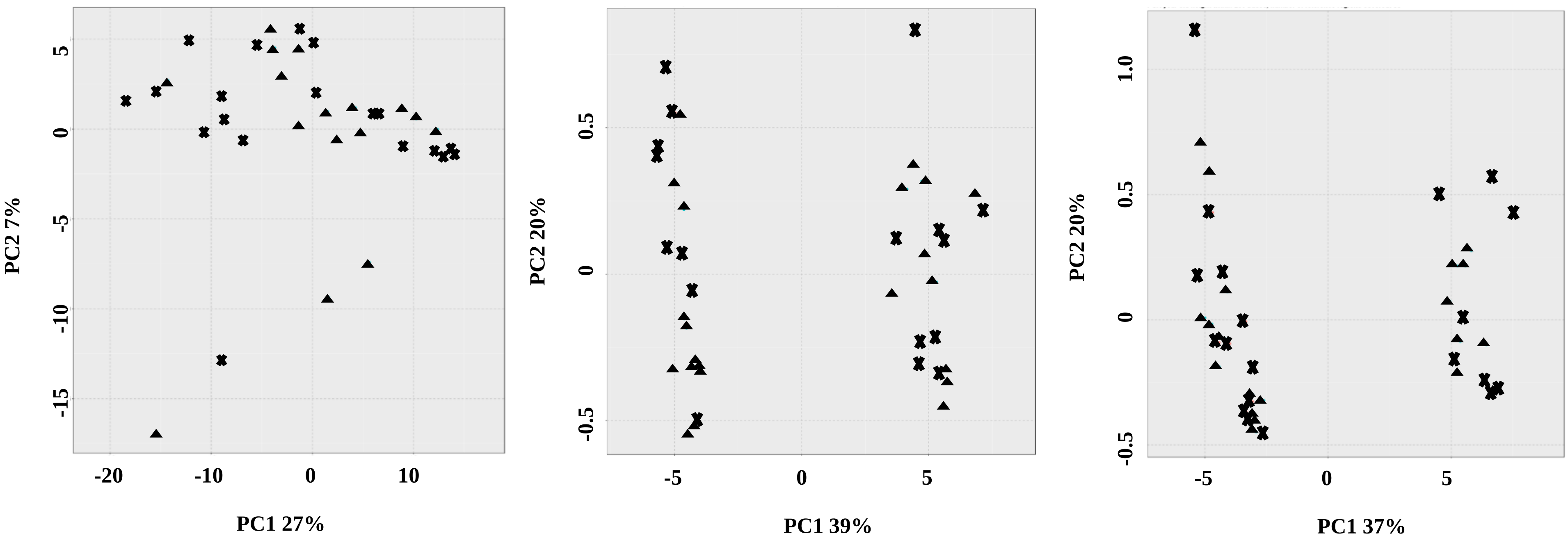

A: PCA cord blood birthweight within 200 bases: 14993 reference regions covered

B: PCA year old birthweight within 200 bases: 33 reference regions covered

C: PCA year old length for age within 200 bases: 33 reference regions covered

Supplementary Figure 9 PCA Plots of Methylation Values for Cell Type Specific CpG Loci

PCA plots based on methylation values for cell type specific CpG loci covered by the sequence capture datasets for experimental groups using 200 base pair intervals around the Illumina EPIC 850K cord and Infinium 450K adult cell specific reference CpG panels. Of the 215000 cord blood CpGs, 14993 DMR reference regions were tested. Of the 450 adult blood CpGs, 33 DMR reference regions (representing CD4 and CD8 lymphocytes, NK cells, neutrophils B-cells and monocytes) were tested. The small number of probes from the adult blood reference panel present in the Methylseq capture DNA sequence dataset does show separation of individual samples into two groups indicating inter-individual differences in cellular composition. However, in all three plots the variation between samples captured in PC1 and PC2 is distributed fairly uniformly across both experimental groups (high or l ow birthweight or tall or short length for age) indicating little difference in cellular composition between groups to confound the determination of differential methylation values. A=cord blood high and low birthweight groups tested with EPIC cord blood reference panel, B=12 month infant blood high and low birthweight groups tested with the Infinium HM450K adult blood panel , C=12 month infant blood tall and short length for aged groups tested with the Infinium 450K adult blood reference panel. The adult blood reference panel was used for the 12 month sample sets in the absence of an age-related 12 month specific reference panel. Triangles= low and crosses= high.
