# Supplementary material for: Identification of methylation changes associated with positive and negative growth deviance in Gambian infants using a targeted methyl sequencing approach of genomic DNA
Source: FASEB Bioadv. 2021 Feb 5;3(4):205–30. doi: 10.1096/fba.2020-00101 (PMC8019263; doi:10.1096/fba.2020-00101)
Supplement: Supplementary file 10 — Table S1 [file FBA2-3-205-s011.pdf]

Supplementary Table 1

Variables plotted by PCA
**Non continuous data**
Birthweight Category
Floor Material
HAZ Category
HCC Category
Season
Sex
Village
Wall Material
**Continuous data**
Birthweight
Birth head circmference
Birthlength
Gestational Age
LAZ Change
HCC level
Maternal Age
Maternal height
Number of Parities
Number of Bedrooms
Crowding Ratio
Number of Animals
Wealth
Delivery Mode
Delivery Problems
Placenta Length
Placenta Width
Placenta Weight
Placenta Depth

Supplementary Table 1 Variables Plotted by Principle Component Analysis

Principal components analysis was used to assess the contribution of a wide range of variables to the variation in the methylseq data.
